# Systematics, biogeography, and character evolution of the legume tribe Fabeae with special focus on the middle-Atlantic island lineages

**DOI:** 10.1186/1471-2148-12-250

**Published:** 2012-12-25

**Authors:** Hanno Schaefer, Paulina Hechenleitner, Arnoldo Santos-Guerra, Miguel Menezes de Sequeira, R Toby Pennington, Gregory Kenicer, Mark A Carine

**Affiliations:** 1 Plant Biodiversity Research, Technische Universität München, Maximus-von-Imhof Forum 2, Freising, D-85354, Germany; 2 Royal Botanic Garden Edinburgh, 20A Inverleith Row, Edinburgh, EH3 5LR, United Kingdom; 3 Unidad de Botánica (ICIA). C/Retama, 2, 38400, Puerto de La Cruz, Tenerife, Islas, Canarias, Spain; 4 Universidade da Madeira, Centro de Ciências da Vida, Funchal, Madeira, Portugal; 5 Plants Division, Department of Life Sciences, The Natural History Museum, Cromwell Road, London, SW7 5BD, United Kingdom

**Keywords:** *Lathyrus*, Legumes, Lentil, Long-distance dispersal, Macaronesia, Pea, *Pisum*, *Vicia*

## Abstract

**Background:**

Tribe Fabeae comprises about 380 legume species, including some of the most ancient and important crops like lentil, pea, and broad bean. Breeding efforts in legume crops rely on a detailed knowledge of closest wild relatives and geographic origin. Relationships within the tribe, however, are incompletely known and previous molecular results conflicted with the traditional morphology-based classification. Here we analyse the systematics, biogeography, and character evolution in the tribe based on plastid and nuclear DNA sequences.

**Results:**

Phylogenetic analyses including c. 70% of the species in the tribe show that the genera *Vicia* and *Lathyrus* in their current circumscription are not monophyletic: *Pisum* and *Vavilovia* are nested in *Lathyrus*, the genus *Lens* is nested in *Vicia*. A small, well-supported clade including *Vicia hirsuta*, *V. sylvatica*, and some Mediterranean endemics, is the sister group to all remaining species in the tribe. Fabeae originated in the East Mediterranean region in the Miocene (23–16 million years ago (Ma)) and spread at least 39 times into Eurasia, seven times to the Americas, twice to tropical Africa and four times to Macaronesia. Broad bean (*V. faba*) and its sister *V. paucijuga* originated in Asia and might be sister to *V. oroboides*. Lentil (*Lens culinaris* ssp. *culinaris*) is of Mediterranean origin and together with eight very close relatives forms a clade that is nested in the core *Vicia*, where it evolved c. 14 Ma. The *Pisum* clade is nested in *Lathyrus* in a grade with the Mediterranean *L. gloeosperma*, *L. neurolobus*, and *L. nissolia*. The extinct Azorean endemic *V. dennesiana* belongs in section Cracca and is nested among Mediterranean species. According to our ancestral character state reconstruction results, ancestors of Fabeae had a basic chromosome number of 2n=14, an annual life form, and evenly hairy, dorsiventrally compressed styles.

**Conclusions:**

Fabeae evolved in the Eastern Mediterranean in the middle Miocene and spread from there across Eurasia, into Tropical Africa, and at least seven times to the Americas. The middle-Atlantic islands were colonized four times but apparently did not serve as stepping-stones for Atlantic crossings. Long-distance dispersal events are relatively common in Fabeae (seven per ten million years). Current generic and infrageneric circumscriptions in Fabeae do not reflect monophyletic groups and should be revised. Suggestions for generic level delimitation are offered.

## Background

**Figure 1:**
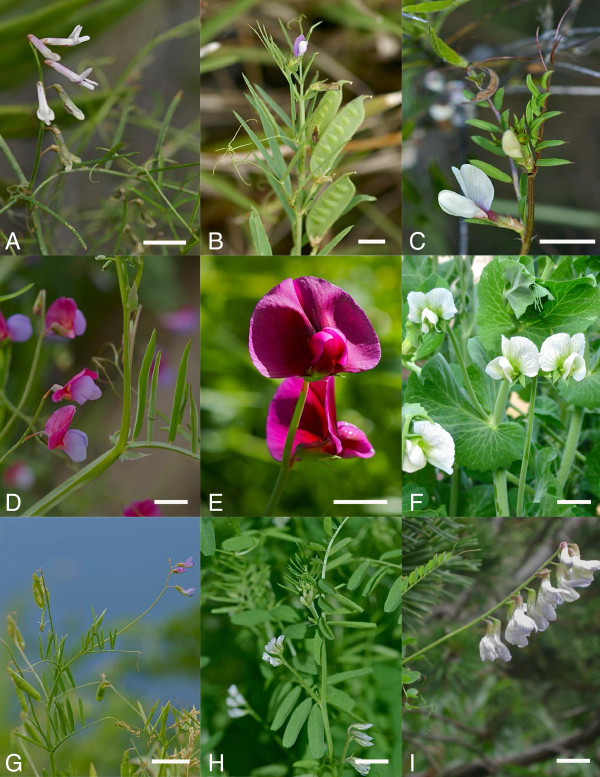
**Examples of morphological diversity in Fabeae.A**) *Vicia cirrhosa*, Gran Canaria, Canary Islands, Spain; **B**) *Vicia bithynica*, Pico, Azores, Portugal; **C**) *Vicia lutea*, Gran Canaria, Canary Islands, Spain; **D**) *Lathyrus angulatus*, Gran Canaria, Canary Islands, Spain; **E**) *Lathyrus tingitanus*, Santa Maria, Azores, Portugal; **F**) *Pisum sativum* ssp. *sativum*, Washington D.C., USA (cultivated); **G**) *Vicia tenuissima*, Flores, Azores, Portugal; **H**) *Vicia hirsuta*, Cambridge, USA (invasive); **I**) *Vicia sylvatica*, Tegernsee, Bavaria, Germany (scale bar: 5 mm; all pictures © H. Schaefer).

Legumes (Fabaceae/Leguminosae) are grown on 12-15% of the world’s arable surface and account for c. 27% of the world’s primary crop production [1]. The tribe Fabeae Rchb. (not Vicieae (Bronn) DC., nom. illeg.) in particular, a group of five genera and c. 380 species ([2,3], our Additional file 1: Annex 1, Figure 1 for some examples) contains several important crop species. These include the pea (*Pisum sativum* L., Figure 1F), lentil (*Lens culinaris* Medik.), broad bean (*Vicia faba* L.), and bitter vetch (*Vicia ervilia* Willd.) that, together with wheat, barley and flax are considered the founder crops of Neolithic agriculture in the Fertile Crescent of Western Asia [4-6]. In addition to crops of worldwide importance, the tribe also contains minor crop species (e.g., *Lathyrus sativus* L., *L. sphaericus* Retz.), forage or green manure species (e.g., *Vicia sativa* L., *V. villosa* L.) and popular ornamentals (e.g. *L. odoratus* L.).

Fabeae have an almost worldwide distribution: of the five genera in the tribe, *Vicia* L. (c. 216 species) and *Lathyrus* L. (c. 150 species) are both most diverse in the Eastern Mediterranean but extend throughout Europe, and both have species in Asia, Northern to Tropical Africa, and North and South America [7,8]. *Vicia* has also colonized Hawaii in the Pacific [9] and the middle-Atlantic archipelagos of the Canaries, Madeira and Azores. The remaining three genera are small and more localized: *Lens* Medik., with 4–8 species, and *Pisum* L. with perhaps two species and several subspecies are both most diverse in the Eastern Mediterranean region; the genus *Vavilovia* Al.Fed. with one or two species is native to the West Asian mountains [2,10]. Fabeae are absent from most of the lowland Tropics and have been introduced to Australia and Polynesia only very recently with European settlers.

While our knowledge of the systematics and biogeography of the Fabaceae is increasing steadily, there are still remarkable gaps. For the tribe Fabeae, the last detailed and comprehensive morphology-based systematic revision was produced by Alefeld [11], who divided the group in two tribes ‘Viciidae’ and ‘Orobidae’ with, collectively, 25 genera. The currently used infrageneric classifications in *Vicia* and *Lathyrus* largely follow the systems suggested by Kupicha [7,8]. For *Lathyrus*, Kupicha accepted 13 sections [8], which have subsequently been modified by Asmussen & Liston [12] and more recently by Kenicer et al. [13], who accepted eleven sections. In *Vicia*, Kupicha [7] introduced two subgenera, *Vicia* and *Vicilla*, and 22 sections with an additional four sections later introduced by Maxted [14]. One species was placed in a separate genus *Anatropostylia*[15] but has also been treated as a monotypic section Anatropostylia of *Vicia*[16]*.*

Recent molecular phylogenetic studies have focussed on sections or genera. Thus, studies on *Vicia* included up to 55 species [17-23]; phylogenetic studies on the genus *Lathyrus* included up to 53 species [12,13,24-26]; several studies analysed the genera *Lens*[27-29] and *Pisum*[30-33]; finally, Oskoueiyan et al. [10] analysed the phylogenetic position of *Vavilovia*. Full chloroplast genomes have recently become available for *Lathyrus sativus* and *Pisum sativum*[34]. In contrast to this growing body of analyses at the generic and sectional level, there are few studies dealing with the entire tribe. The only exceptions are family-wide or multi-tribe legume phylogenetic studies, which included a limited sample of species across Fabeae (e.g., [35-39]). Those studies suggested that Fabeae is monophyletic and sister group to the clover genus *Trifolium* L. Even though sampling of Fabeae in those family-wide studies has been limited, they suggest that plastid DNA sequence data do not agree with morphology-based genus circumscriptions in the tribe and that *Vicia* might be paraphyletic [36]. Altogether, these studies highlight the need of a comprehensive tribal-level study of Fabeae.

Several hypotheses have been proposed to explain the worldwide distribution of Fabeae but there has been no comprehensive biogeographic analysis of the entire tribe. Simola [40] suggested a South American origin of *Lathyrus* followed by a dispersal event to Africa and then into the Mediterranean. In contrast, Kupicha [8] suggested that *Lathyrus* and *Vicia* originated at high latitudes in the Old World, and might have migrated later to the Mediterranean and to North America via Greenland or from Asia via Beringia to Alaska. From North America, the lineages could have spread into South America in the late Tertiary. A North American origin of the South American Notolathyrus group was also suggested by Burkart [41] and Asmussen & Liston [12]. Based on DNA sequence data, Kenicer et al. [13] suggested an eastern Mediterranean origin for *Lathyrus* followed by range expansion into northern Eurasia. The Beringian land bridge would then have allowed migration into North America. According to Kenicer et al. [13], the most likely origin of the South American *Lathyrus* species is directly from Eurasia via long-distance dispersal (sea currents). In this context the question arises whether those lineages used stepping-stone islands in the middle-Atlantic as suggested by Axelrod [42] and more recently by Fernández-Palacios et al. [43].

**Figure 2:**
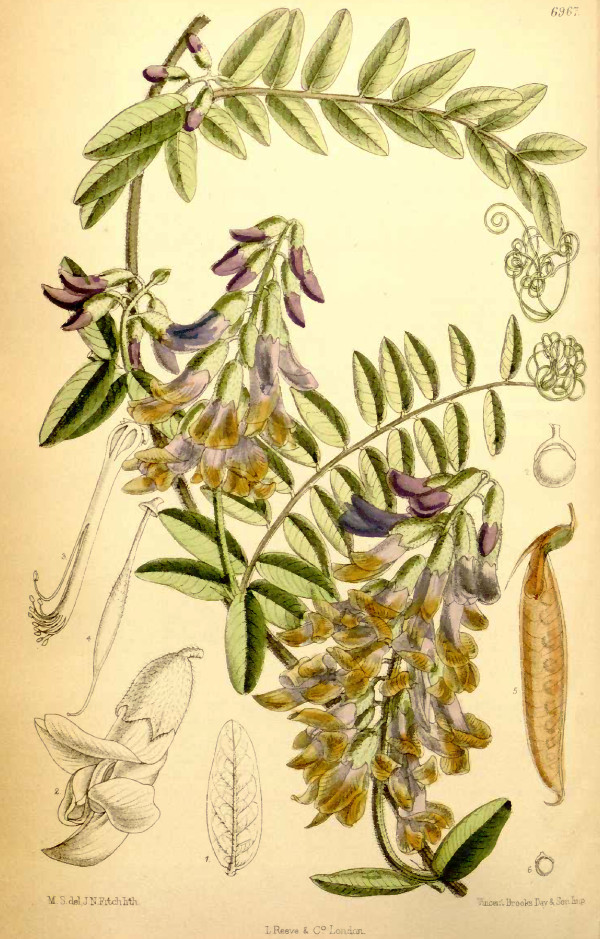
***Vicia dennesiana *H. C. Watson.** The extinct Azorean endemic *Vicia dennesiana*, here shown to be nested in the Mediterranean section Cracca. This drawing originally published in Curtis’s Botanical Magazine in 1887, ser. 3, vol. 43, was made of a plant cultivated at Royal Botanical Gardens Kew from seeds of plants cultivated in the private garden of H. C. Watson, who obtained his seeds from T. C. Hunt, then British consul on São Miguel Island, Azores [47]. The Kew plants were subsequently lost in a late May frost [48] and the species was not rediscovered ever since.

The stepping-stone hypothesis is supported by the existence of forty-five species of Fabeae on the volcanic oceanic Atlantic islands of which nine are currently considered as endemic to one or more of the islands. On Iceland in the extreme North, five species are possibly native (*Lathyrus japonicus* Willd., *L. palustris* L., *L. pratensis* L., *Vicia cracca* L. and *V. sepium* L.) and four occur as casuals (*V. angustifolia* L.*, V. hirsuta* (L.) Gray, *V. sativa*, *V. villosa*) [44]. Further to the South, in the Azores archipelago, a total of 14 *Vicia*, 10 *Lathyrus*, and *Lens culinaris* have been reported [45,46]. All are considered introduced [45], with the exception of *Vicia dennesiana* H. C. Watson (Figure 2), which is endemic to the Azorean island of São Miguel but has not been seen since the mid 19th century [45,47,48]. For Madeira, 15 *Vicia*, 11 *Lathyrus*, and *Lens culinaris* have been reported [49]. Of those, three species are endemic to Madeira (*V. capreolata* Lowe, *V. costae* A. Hansen and *V. ferreirensis* Goyder from Porto Santo), 15 others considered native, and nine are probably introduced [49]. In the Canary Islands, 22 *Vicia*, 12 *Lathyrus*, two *Lens*, and one *Pisum* species have been found [50]. Five species are currently considered to be endemic to the Canaries: *Vicia chaetocalyx* Webb & Berthel., *V. cirrhosa* C. Sm. ex Webb & Berthel. (Figure 1A), *V. filicaulis* Webb & Berthel., *V. nataliae* U. Reifenb. & A.Reifenb., and *V. scandens* R.P.Murray. Of the remainder 13 are considered at least possibly native, and 19 are very likely recent introductions [50]. No native Fabeae have been reported from the Selvagens or, further south, from Cape Verdes, St. Helena, or Tristan da Cunha.

The aims of this study were to examine the evolution, biogeography and classification of Fabeae. Newly generated plastid and nuclear DNA sequence data (*rbcL*, *matK*, *trnL-trnF*, *trnS-trnG*, *psbA-trnH*, and ITS (internal transcribed spacer) region) for Fabeae species from the middle-Atlantic islands, North and South America, Africa, and Eurasia are analysed in conjunction with previously published data to establish a phylogeny of the tribe that includes c. 70% of the accepted species (often more than one accession per taxon), with representatives sampled from all sections and all geographic regions in which the tribe occurs. This phylogenetic framework is used to (i) test current morphology-based generic and infrageneric circumscriptions and (ii) analyse the evolution of characters used for generic and infrageneric delimitation, notably stylar and life form characters [7,8]. Using a Bayesian relaxed molecular clock approach and ancestral range reconstruction, we further test the hypothesis of a Eurasian origin followed by dispersal to the Americas (a) across the Atlantic or (b) via land bridges or ‘stepping stone’ islands.

## Results

### Phylogenetic analyses

**Figure 3:**
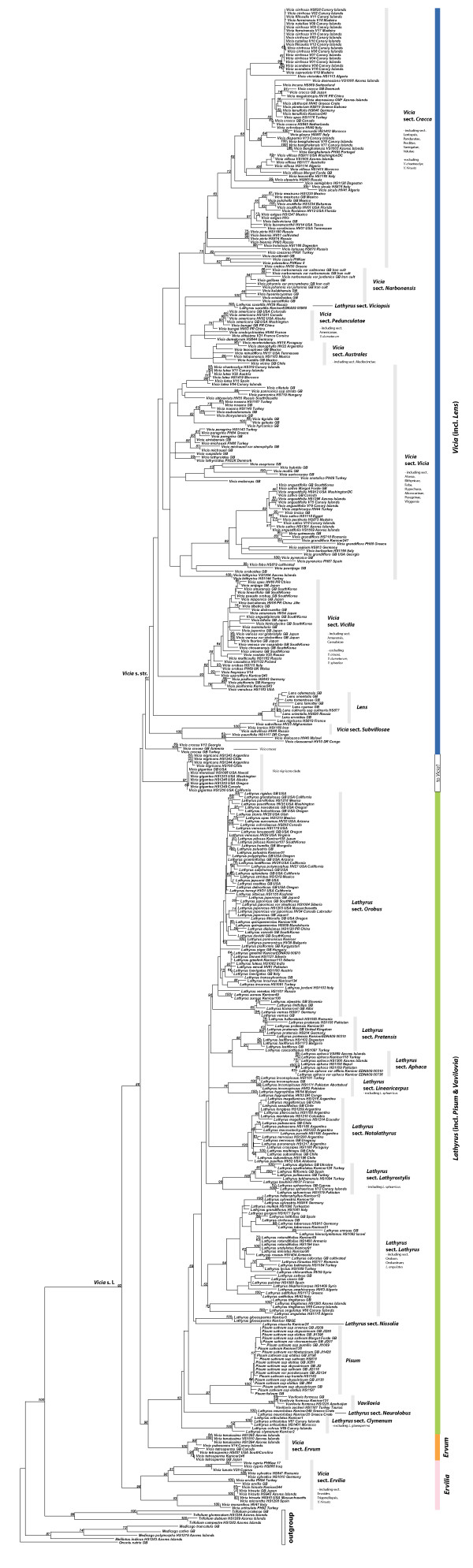
**Maximum likelihood phylogeny of the Fabeae.** Best maximum likelihood tree based on the combined chloroplast and ITS dataset for 470 ingroup accessions (262 species) plus seven outgroups (6200 aligned nucleotides plus 83 gap characters). Likelihood bootstrap values ≥ 50% are given at the nodes. The five currently accepted genera in the tribe plus the proposed *Ervum* and *Ervilia* and the sections [7,8,14] are indicated.

Maximum likelihood (ML) and Bayesian analyses of chloroplast (*rbcL*, *matK*, *trnL/trnL-trnF*, *trnS-trnG*, *psbA-trnH*) and nuclear ribosomal ITS sequence data for 262 of the c. 380 species currently accepted in the tribe (Additional file 2: Table S1, Additional file 1: Annex 1) yield a generally well-resolved and statistically supported phylogeny although some nodes of interest are only weakly or not supported (see Figure 3 for ML phylogeny of combined data, and Additional file 3: Figure S1-S7 Additional file 4: Figure S2, Additional file 5: Figure S3, Additional file 6: Figure S4, Additional file 7: Figure S5, Additional file 8: Figure S6, Additional file 9: Figure S7 for phylogenies from individual markers; Additional file 10: Figure S8 for Bayesian phylogeny from combined data). The nuclear ribosomal ITS (Additional file 3: Figure S1) and combined plastid (S7) phylogenies are congruent (based on comparison of clades with BS ≥ 65%). We thus find no evidence for a strong effect of hybridization in the evolution of the Fabeae and analyses of a combined matrix is justified.

Our results confirm the monophyly of *Lens* and *Pisum* (99 and 100% BS respectively, Figure 3; 1.0 PP, Figure S8) and the paraphyly of *Vicia*. In contrast to previous studies, we find that *Lathyrus* is not monophyletic and includes *Pisum* and *Vavilovia* (Figure 3; Additional file 10: Figure S8). Currently recognized subgenera in *Vicia* and sections with more than one species in both *Vicia* and *Lathyrus* are mostly not recovered as monophyletic groups (see Table 1). Of the 13 sections in *Lathyrus*, sections Neurolobus, Nissolia, Orobon, Orobastrum, and Viciopsis are monotypic. Our sampling strategy allowed us to test the monophyly of seven of the eight sections of *Lathyrus* that comprise multiple species. Only Notolathyrus and Pratensis are found to be monophyletic (100 and 88% BS respectively, Figure 3; 1.0 PP, Additional file 10: Figure S8). We had material of only one of the two species in section Aphaca but since the two species are distinguishable only through slight differences in style shape and fruit morphology, section Aphaca is very likely also monophyletic. Sections Orobon and Orobastrum are both nested in section Lathyrus. Section Linearicarpus is polyphyletic: *L. hygrophilus* Taub. and *L. inconspicuus* L. group together (76% BS, Figure 3; 1.0 PP, Additional file 10: Figure S8), whereas *L. angulatus* L. (Figure 1D) belongs to section Lathyrus (70% BS, Figure 3; 1.0 PP, Additional file 10: Figure S8) and *L. sphaericus* groups with section Lathyrostylis (99% BS, Figure 3; 1.0 PP, Additional file 10: Figure S8). *Lathyrus gloeosperma* Warb. & Eig from Kupicha’s section Clymenum groups with *L. nissolia* L. in the ML reconstruction (but <50% BS, Figure 3). *Lathyrus saxatilis* (Vent.) Vis. (representing Kupicha’s monotypic section Viciopsis) is placed in the core *Vicia* and may be sister to a clade that includes *V. dumetorum* L. and *V. americana* Muhl. ex Willd. although its closest relatives in *Vicia* are unclear due to low support values (Figure 3; Additional file 10: Figure S8).

**Table 1:** Overview of morphology-based sections in Fabeae compared to molecular results.

**Genus**	**Subgenus**	**Section**	**Geographic range**	**No. of accepted species**	**No. of sequenced species**	**Monophyletic?**
*Lathyrus*		Aphaca	Eurasia, Mediterranean region	2	1	?
		Clymenum	Mediterranean region	4	3	No
		Lathyrus	Eurasia	31	15	No
		Lathyrostylis	Eurasia	16	6	No
		Linearicarpus	Eurasia, Mediterranean region	7	3	No
		Neurolobus	Crete	1	1	NA
		Nissolia	Eurasia, Mediterranean region	1	1	NA
		Notolathyrus	S America, Southern N America	22	14	Yes
		Orobastrum	Eurasia, Mediterranean region	1	1	NA
		Orobon	Caucasus Mts.	1	1	NA
		Orobus	Eurasia, Mediterranean region, N & C America	44	36	No
		Pratensis	Eurasia, Mediterranean region	6	2	Yes
		Viciopsis	S Europe, Turkey, NW Africa	1	1	NA
*Lens*			Mediterranean region, Asia Minor	5	5	Yes
*Pisum*			Mediterranean region, Asia Minor	2	2	Yes
*Vavilovia*			Caucasus Mts.	2	2	Yes
*Vicia*	Vicia	Atossa	Eurasia, Mediterranean region	4	2	No
		Faba	Eurasia, Mediterranean region	2	2	Yes
		Bithynicae	Mediterranean region	1	1	NA
		Wiggersia	Eurasia, Mediterranean region	2	2	No
		Microcarinae	Mediterranean region	1	1	NA
		Narbonensis	Mediterranean region, Asia Minor	7	7	Yes
		Hypechusa	Eurasia, Mediterranean region	13	11	No
		Peregrinae	Mediterranean region, Asia Minor	3	3	Yes
		Vicia	Eurasia, Mediterranean region	5	5	No
	Vicilla	Americanae	East Asia, N America	2	2	Yes
		Anatropostylia	Asia	1	0	NA
		Australes	Mexico, C & S America	17	6	No
		Cassubicae	Eurasia, Mediterranean region, Pacific coast of N & S America	7	7	No
		Cracca	Eurasia, Mediterranean region, N America	45	29	No
		Ervilia	Mediterranean region, Asia Minor	2	1	?
		Ervoides	Mediterranean region, Asia Minor	1	1	NA
		Ervum	Eurasia, Mediterranean region	3	3	Yes
		Lentopsis	Turkey	1	1	NA
		Mediocinctae	Southern USA	1	1	NA
		Panduratae	Mediterranean region, Asia Minor	3	2	No
		Pedunculatae	S Europe, Asia Minor, NW Africa	3	2	No
		Perditae	Azores	1	1	NA
		Subvillosae	Asia	1	1	NA
		Trigonellopsis	Mediterranean region, Asia Minor	3	2	Yes
		Variegatae	S Europe, Caucasus Mts., Asia	3	1	?
		Vicilla	Eurasia, Mediterranen region	15	13	No
		Volutae	Southeastern Europe, Caucasus Mts.	1	1	NA

List of the sections based on Kupicha’s system [7,8] and Maxted’s modifications in *Vicia*[14] with total species number, number of species included in our analyses and status as monophyletic or not based on the molecular results.

Within *Vicia* s.str., most of the species of Kupicha’s subgenus *Vicia* group together, with the notable exception of *Vicia narbonensis* L. and close relatives (section Narbonensis sensu Maxted) from the Eastern Mediterranean and Asia Minor. The second subgenus, *Vicilla*, is clearly polyphyletic (see below; Figure 3). Nine of the 27 sections currently recognized in *Vicia* are monotypic: sections Anatropostylia, Bithynicae, Ervoides, Lentopsis, Mediocinctae, Microcarinae, Perditae, Subvillosae, and Volutae (Table 1). Our sampling strategy allowed us to test the monophyly of 16 of the 18 remaining sections. Of these, sections Americanae, Ervum, Faba, Narbonensis, Peregrinae, and Trigonellopsis are found to be monophyletic. Section Cracca is not monophyletic because (i) sections Lentopsis, Panduratae, Perditae, Variegatae, and Volutae are nested in a clade otherwise comprising species from this section and (ii) *Vicia chaetocalyx* and *V. hirsuta* that were previously assigned to this section are placed outside the Cracca clade (Figure 3). With regards to section Vicilla*, V. americana*, *V. bungei* Ohwi, *V. crocea* (Desf.) B. Fedtsch., *V. dumetorum*, *V. gigantea*, and *V. nigricans* are not resolved with other members of the section. These species are resolved as paraphyletic with respect to sections Cassubicae and Amurense. Section Australes is shown to be paraphyletic with respect to section Mediocinctae and section Pedunculatae includes section Americanae and *V. dumetorum* (Figure 3). Section *Vicia* is also not monophyletic because sections Atossa, Hypechusa, Peregrinae, and Faba are nested within it. *Vicia* section Ervum is sister to all *Lathyrus* species (except *L. saxatilis*) plus *Pisum* and *Vavilovia* (98% BS, Figure 3; 1.0 PP, Additional file 10: Figure S8). *Vicia* sections Ervilia, Ervoides, and Trigonellopsis group together (98% BS, Figure 3; 0.99 PP, Additional file 10: Figure S8) and form a clade that is sister to all other Fabeae (95% BS, Figure 3; 1.0 PP, Additional file 10: Figure S8).

At the species level, our material of the Macaronesian endemic *Vicia chaetocalyx* is nested in a highly supported clade of *V. lutea* L. accessions (99% BS, Figure 3). There is no resolution in the *V. cirrhosa* group from the Canaries and Madeira in any of the analyses (Figure 3, Additional file 3: Figure S1-S7, Additional file 4: Figure S2, Additional file 5: Figure S3, Additional file 6: Figure S4, Additional file 7: Figure S5, Additional file 8: Figure S6, Additional file 9: Figure S7). In contrast, the *V. sativa* group is genetically quite heterogeneous, especially in nuclear ITS sequences (Additional file 3: Figure S1) and while *V. sativa* s.l. is monophyletic, the different accessions of *V. angustifolia* and *V. sativa* s.str. do not group together. In the *V. tetrasperma* lineage, *V. tenuissima* Schinz & Thell. and *V. pubescens* (DC.) Link are genetically distinct from *V. tetrasperma* (L.) Schreb. s. str. both in ITS (Additional file 3: Figure S1) and combined plastid sequences (Additional file 9: Figure S7). Nuclear ITS sequence differences also support the separation of North and South American populations of *V. nigricans* s. l. as *V. gigantea* Hook. and *V. nigricans* Hook. & Arn. s. str. respectively (Additional file 3: Figure S1). Heterogeneity at the genetic level is also evident for the *Lathyrus aphaca* L. clade, a morphologically variable taxon widespread from the Macaronesian islands to the Himalayas (Figure 3; Additional file 3: Figure S1; Additional file 9: Figure S7). In contrast, *Lathyrus undulatus* Boiss. and *L. miniatus* Steven do not appear to be genetically distinct from *L. rotundifolius* Willd. s. str. and the Romanian endemic *L. hallersteinii* Baumg. is nested in a highly supported clade of *L. pratensis* accessions (99% BS, Figure 3).

#### Molecular dating and ancestral range reconstruction

**Figure 4:**
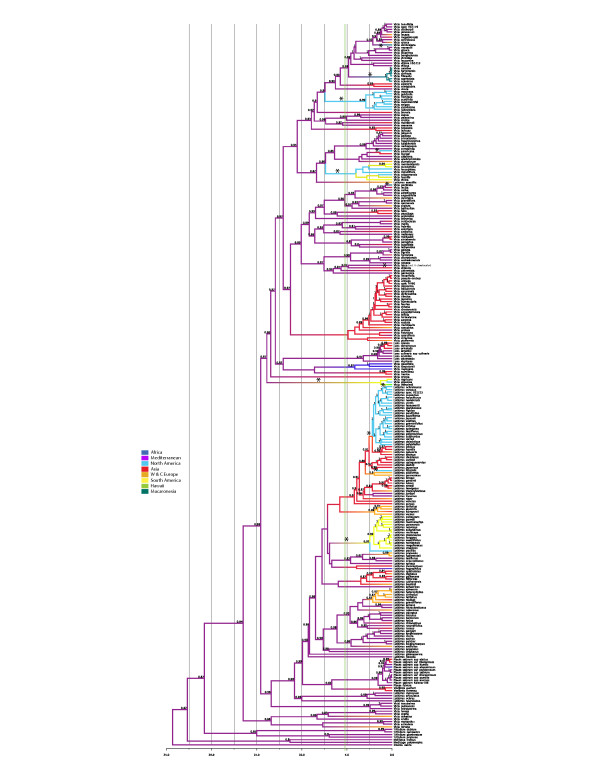
**Biogeographic history of the Fabeae.** Reconstruction of the biogeographical history of the tribe Fabeae using BEAST and the approach of Lemey et al. [79]. Posterior probability values for the range reconstructions are given at the nodes. Asterisks indicate LDD events. Geographical regions are colour-coded: Mediterranean - purple, Central and Western Europe - orange, Asia - red, Hawaii - green, Macaronesia - turquoise, Africa - dark blue, North America - light blue, South America - yellow. The vertical light green bar marks the first opening of the Bering Strait c. 5.4-5.5 Ma [57].

**Figure 5:**
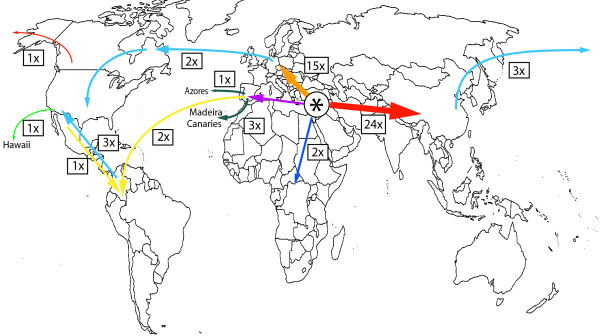
**Overview map of the biogeographic history of Fabeae.** World map showing the reconstructed biogeographical scenario for the Fabeae: origin 23–16 Ma in the Mediterranean, then at least 24 times range expansion into Asia and 15 times into Central and Western Europe. Furthermore, at least two range expansions or dispersal events into tropical Africa. South America was colonized twice via LDD from the Mediterranean and once via range expansion from North America. North America was colonized once via LDD from Europe/Mediterranean, three times from Asia and perhaps three times through range expansion of South American lineages. Macaronesia was reached four times by LDD from the Mediterranean with one colonisation of the Azores and three of the Canaries/Madeira.

The results of our Bayesian molecular clock and ancestral range analyses suggest a Mediterranean origin and a crown age of c. 23–16 Myr for Fabeae, with a best estimate for the associated substitution rate of 0.0045 substitutions per site per Myr (subst/site/My) (Figure 4; Additional file 11: Figure S9). We reconstruct a minimum of three dispersal events to the middle-Atlantic islands (four if *V. chaetocalyx* is accepted) and seven to the Americas. None of the New World lineages originates from the Atlantic islands (Figures 4, 5). The oldest Macaronesian lineage, the *Vicia scandens* clade, split from a Mediterranean ancestor 4.9-2.4 Ma, 0.0019 subst/site/My (Additional file 11: Figure S9) and colonized the Canary Islands and Madeira plus Porto Santo (crown age 0.9-0.15 Ma, 0.0026 subst/site/My). The second dispersal led to the establishment of the *Vicia dennesiana* lineage in the Azores. The age estimate for this event is not very robust due to the low resolution in the *V. cracca* clade, where *V. dennesiana* is placed but it occurred probably more recently than the Canary island lineage, between 2.9-1.1 Ma, 0.0028 subst/site/My (Figure 4, Additional file 11: Figure S9). The remaining colonization events are even more recent: the Madeiran species or form *Vicia pectinata* Lowe split from the Mediterranean *Vicia sativa* 0.8-0.1 Ma (0.002 subst/site/My), whereas the Canary island *Vicia chaetocalyx* is nested in a clade of the mainly Mediterranean *Vicia lutea* (Figure 1C) and might have arrived only with the first human settlers.

We reconstruct seven migration events to the New World: (1) the *Vicia nigricans* clade split from the Eurasian *Vicia* lineages 13.7-8.5 Ma (0.0014 subst/site/My). This split is reconstructed as a long distance dispersal (LDD) event from the Mediterranean region to South America with high support (0.97 PP, Figure 4) but the *Vicia nigricans* clade might also be sister to the Caucasian *V. crocea* (Additional file 11: Figure S9), which would indicate dispersal from Asia via Beringia. The ancestor of the Hawaiian endemic *Vicia menziesii* Spreng. then dispersed from Western America to Hawaii 2.5-0.1 Ma, 0.0014 subst/site/My (Additional file 11: Figure S9); (2) the North American *Vicia ludoviciana* Nutt. clade split from a Mediterranean ancestral lineage 8.9-6.5 Ma and dispersed across the Atlantic, possibly floating in surface water (Figure 4; Additional file 11: Figure S9); (3) the mainly South American *Vicia* section Australes (incl. *V. leucophaea* Greene) split from a Mediterranean ancestral lineage 8.5-4.8 Ma (0.0016 subst/site/My) and is again most likely a case of LDD across the Atlantic (Figure 4; Additional file 11: Figure S9); (4) the South American section Notolathyrus split from a Mediterranean ancestral lineage 8.6-6.1 Ma (0.0018 subst/site/My), representing the third LDD event in the same time window of 9–5 Ma (Figure 4; Additional file 11: Figure S9); (5) the large clade of North American species in *Lathyrus* section Orobus split from an Asian ancestral lineage 2.9-1.8 Ma, 0.0017 subst/site/My (Figure 4; Additional file 11: Figure S9); (6) colonization of the North American coasts by the *Lathyrus littoralis*/*L. japonicus* lineage started c. 3–1.7 Ma, 0.0017 subst/site/My (Figure 4; Additional file 11: Figure S9); (7) and finally the split between the North American *Vicia americana* and its Asian sister *V. bungei* is dated to 2.3-0.2 Ma, 0.002 subst/site/My (Additional file 11: Figure S9).

#### Origin of the crop species in Fabeae

The pea, *Pisum sativum* s. l. (including *P. elatius* M.Bieb. and *P. humile* Mill.) is sister to the Eastern Mediterranean *Pisum fulvum* Sibth. & Sm. (100% BS, Figure 3; 1.0 PP, Additional file 10: Figure S8) and both are sister to *Vavilovia formosa* (Stev.) Fed. (≡ *Pisum formosum* (Stev.) Alef.) and *V. aucheri* (Jaub. & Spach) Fed. (≡ *Pisum aucheri* Jaub. & Spach) from the Caucasus Mountains (98% BS, Figure 3; 1.0 PP, Additional file 10: Figure S8). The crown age of the *Pisum* clade is estimated to 2.3-0.8 Ma (Additional file 11: Figure S9); the divergence between *Pisum* and *Vavilovia* dates back to 9.8-4.8 Ma (Additional file 11: Figure S9). Lentil, *Lens culinaris* ssp. *culinaris*, is nested among a group of very closely related taxa. Their mostly allopatric distribution, morphological similarity and very recent divergence estimates suggest that they might be best treated as subspecies or varieties of *Lens culinaris*. All together are sister to the Mediterranean *Lens nigricans* (M.Bieb.) Godr. (99% BS, Figure 3; 1.0 PP, Additional file 10: Figure S8). The split between the *L. culinaris* group and *L. nigricans* occurred 4.9-1.9 Ma, whereas the stem lineage of *Lens* might have split from its *Vicia* ancestors 14.9-12.6 Ma. Faba bean or broad bean, *Vicia faba* is sister to the Himalayan *V. paucijuga* B. Fedtsch., which is often treated as a subspecies of *V. faba*. Both are nested in section Vicia (incl. sect. Atossa, Bithynicae, Hypechusa, Microcarinae, Peregrina, and Wiggersia) with an estimated stem age of 8–3.8 Ma but this is not robust due to the lack of a well supported placement of the lineage in any of our phylogeny estimates (Figure 3; Additional file 10: Figure S8, Additional file 12: Figure S10). The ancient crop species *Vicia ervilia* is placed in the *Ervilia* clade that is here recovered as the sister group to all other Fabeae. It split from its closest relatives in the *V. sylvatica* L. clade 11.2-5.0 Ma (Additional file 11: Figure S9). *Vicia sativa* belongs in a group of closely related and insufficiently studied Mediterranean taxa. In our ML and Bayesian analyses, the *V. sativa* group (including *V. pectinata*, *V. angustifolia*, *V. incisa* M.Bieb., and *V. amphicarpa* Dorthes) is consistently reconstructed as sister to *V. pyrenaica* Pourr. (but <50% BS, Figure 3; <0.8 PP, Figure S8) from which it split 5.6-2.2 Ma. The economically most important species in the genus *Lathyrus*, the ornamental sweet pea, *L. odoratus*, from southern Italy and Sicily, is sister to the wider Mediterranean *L. hirsutus* L. (94% BS, Figure 3; 1.0 PP, Figure S8), from which it diverged 2.5-0.4 Ma (Figure S9). Finally, the minor crop *L. sphaericus* is sister to the Mediterranean *L. bauhinii* Genty clade (99% BS, Figure 3; 1.0 PP, Additional file 10: Figure S8), from which it diverged 7.6-4.5 Ma (Additional file 11: Figure S9).

#### Character evolution

**Figure 6:**
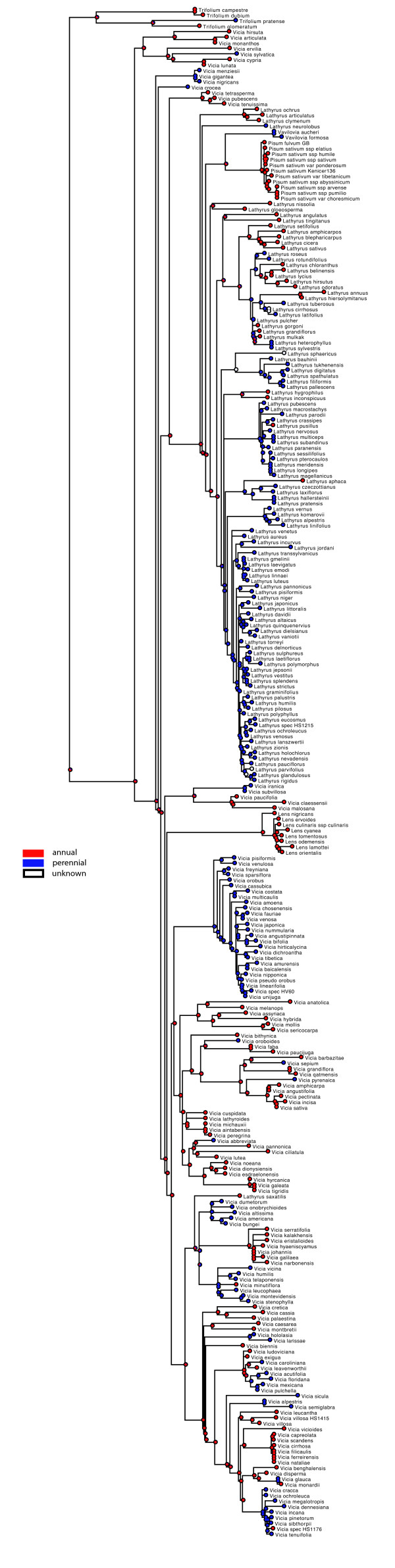
**Life form evolution in Fabeae.** Evolution of life form in Fabeae reconstructed under the Markov k-state one-parameter model on the best maximum likelihood tree using Mesquite ver. 2.75 [82]. Colour code: blue - perennial, red - annual (rarely biannual *Vicia biennis*).

**Figure 7:**
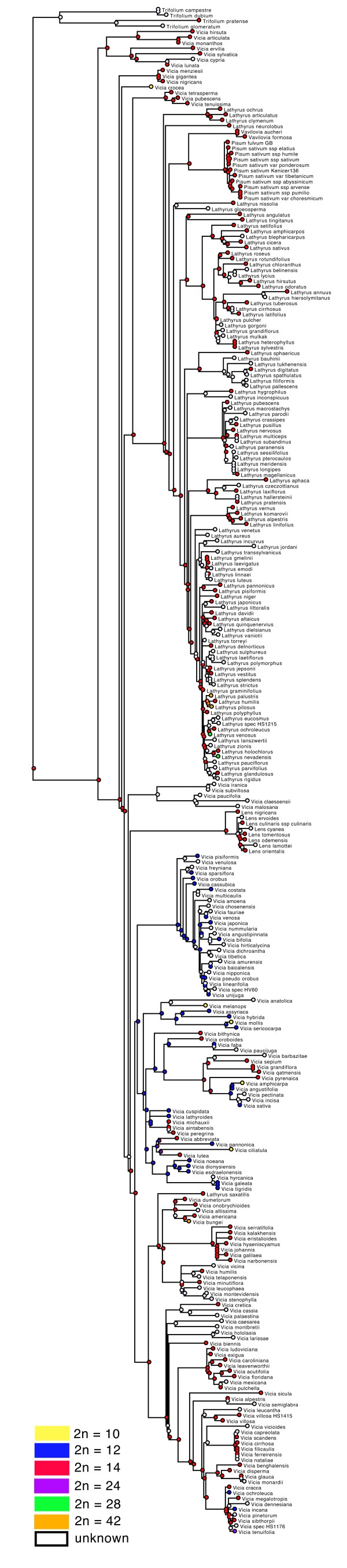
**Evolution of chromosome numbers in Fabeae.** Evolution of chromosome number in Fabeae reconstructed under the Markov k-state one-parameter model on the best maximum likelihood tree using Mesquite ver. 2.75 [82]. Colour code: yellow - 2n=10, blue - 2n=12, red - 2n=14, purple 2n=24, green - 2n=28, orange 2n=42.

**Figure 8:**
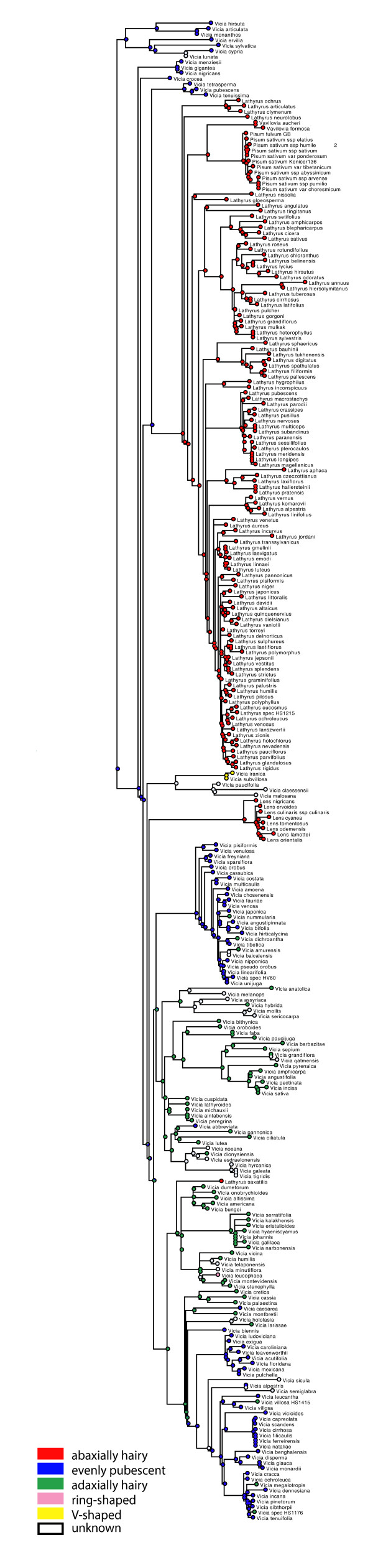
**Evolution of stylar pubescence in Fabeae.** Evolution of stylar pubescence in Fabeae reconstructed under the Markov k-state one-parameter model on the best maximum likelihood tree using Mesquite ver. 2.75 [82]. Colour code: red - abaxially hairy, blue - evenly hairy, green - adaxially hairy, pink - densely hairy ring, yellow - V-shaped hairy zone.

**Figure 9:**
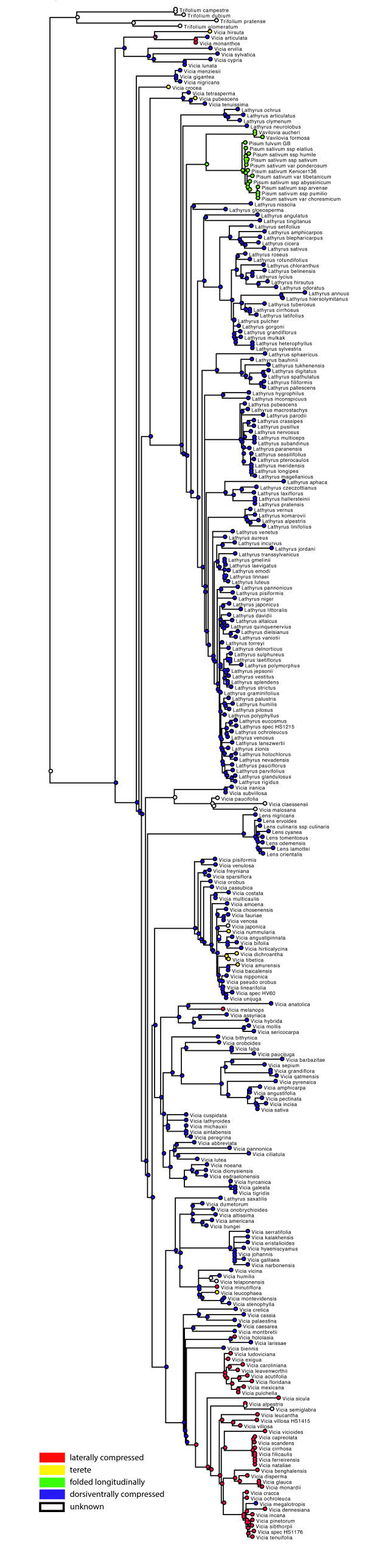
**Evolution of stylar shape in Fabeae.** Evolution of stylar shape in Fabeae reconstructed under the Markov k-state one-parameter model on the best maximum likelihood tree using Mesquite ver. 2.75 [82]. Colour code: red - laterally compressed, yellow - terete, green - folded longitudinally, blue - dorsiventrally compressed.

Annual life form is reconstructed as ancestral in the tribe and a perennial life form evolved at least 20 times independently followed by several reversals to annual life form (Figure 6). The ML ancestral character reconstruction indicates that a basic chromosome number of 2n=14 is most likely for the origin of the tribe (Figure 7). From that, reductions to 2n=12 or 2=10 and duplications to 2n=24, 2n=28, and 2=42 have occurred. Major clades in Fabeae seem to be characterized by common stylar pubescence patterns (Figure 8). From an originally evenly hairy style (still prominent in the *Ervum*, *Ervilia*, and early-branching *Vicia* clades), the *Lathyrus*-*Pisum*-*Vavilovia* clade evolved an adaxially hairy style, whereas some of the more derived *Vicia* clades are dominated by an abaxially hairy style. There are, however, many exceptions: *Lathyrus saxatilis*, for example, with an adaxially hairy style is deeply nested in *Vicia*. The Asian *Vicia subvillosa* (Ledeb.) Boiss. has a unique type of style with V-shaped hairy areas. It is unknown whether this character is shared with its African sister species *V. malosana* (Baker) Baker f., *V. paucifolia* Baker, and *V. claessensii* De Wild. Another unique pattern is found in *V. leucophaea*, which has a densely hairy ring on its style that seems to have evolved from an abaxially hairy style (Figure 8). Style shape, another traditional character for Fabeae classification, varies little within the tribe: most species have a dorsiventrally compressed style, which is also reconstructed as most likely ancestral condition in the tribe (Figure 9). Exceptions are most of the *Vicia* species in section Cracca, which have a laterally compressed style. A terete style (circular in cross section) has evolved six to seven times independently in the tribe: in the Mediterranean *V. pubescens*, the Eurasian *V. hirsuta* and *Vicia crocea,* the North American *V. leucophaea*, and the East Asian *V. amurensis* Oett., *V. tibetica* Prain ex C.E.E. Fisch., *V. nummularia* Hand.-Mazz., and *V. dichroantha* Diels. Furthermore, *Pisum* and *Vavilovia* are characterised by a longitudinally folded style otherwise unknown in the tribe.

## Discussion

### Taxonomic implications

The molecular data show that most of the currently recognized genera and subgenera, as well as many of the traditional sections are not monophyletic. *Pisum* and *Vavilovia* are sister groups and nested in *Lathyrus*, which together with *Lens* is nested in *Vicia*. This grouping is also supported from the (few) currently available whole plastid genomes for Fabeae: *Lathyrus sativus* and *Pisum sativum* share four gene losses (infA, rps16, rpl22, and rpl23) and the loss of the first intron of clpP and the cis-intron of rps12 [34]. *Pisum* and *Lathyrus* furthermore share the phytoalexin pisatin, which was not found in *Vicia* and *Lens*[51]. The two latter genera both had the phytoalexin wyerone in all analysed species except in *Vicia articulata* and *V. ervilia*[52], which we find to be sister to all Vicieae. Thus the presence of wyerone could be a synapomorphy for *Vicia* and *Lens*.

Apart from Alefeld’s concept of splitting the tribe into a multitude of tiny genera [11], the most radical solution to obtain a more natural classification of Fabeae would be the transfer of all species of the four currently accepted smaller genera to a broadly circumscribed genus *Vicia*. This, however, would require more than 100 new combinations. An alternative (and perhaps preferable) solution would be to transfer *Pisum* and *Vavilovia* to a then monophyletic *Lathyrus*. *Vicia* section Ervum (the clade comprising *V. tetrasperma*, *V. tenuissima* (Figure 1G), and *V. pubescens*) could be raised to genus-level; the name *Ervum* L. with the lectotype *E. tetraspermum* L. is available. A monophyletic *Vicia* could then be obtained by transferring *Lens* and *Lathyrus saxatilis* to *Vicia*. The clade comprising *Vicia cypria* Kotschy, *V. lunata* (Boiss. & Bal.) Boiss., *V. ervilia*, *V. hirsuta* (Figure 1H), *V. sylvatica* (Figure 1I), *V. monanthos* (L.) Desf. and *V. articulata* Hornem. (mainly Kupicha’s sections Ervilia, Ervoides, and Trigonellopsis [7] could be split from *Vicia* s. str. and raised to genus-level. Among the available names, *Ervilia* Link seems the most appropriate. Of the species not sequenced, *V. koeieana* Rech.f. (= *Anatropostylia koeieana* (Rech.f.) Kupicha) and *V. quadrijuga* P.H.Davis very likely belong here too, so that a genus *Ervilia* would probably comprise at least nine species.

Most of the sections also need to be revised if they are supposed to reflect monophyletic groupings: section Cracca should include the former sections Lentopsis, Panduratae, Perditae, Variegatae, and Volutae. Section *Vicilla* should be recircumscribed to include sections Amurense and Cassubicae. Section Mediocinctae could be lumped with section Australes. Section Pedunculatae should be merged with section Americanae. Section Vicia could be defined in a broader sense to accommodate sections Atossa, Bithynicae, Faba (sensu Maxted [14]), Hypechusa, Microcarinae, Peregrinae, and Wiggersia. In *Lathyrus*, the clade including *L. alpestris* (Waldst. & Kit.) Kit. ex Reichb. and *L. vernus* (L.) Bernh. could be split from section Orobus. Sections Orobon and Orobastrum could be lumped with section Lathyrus. The circumscriptions of sections Linearicarpus, Lathyrus, and Lathyrostylis all need to be slightly modified in order to obtain monophyletic groups. Newly discovered Fabeae clades that might warrant description of additional sections include (i) the sister group of section Subvillosae, comprising the African *V. claessensii*, *V. malosana*, and *V. paucifolia* (delimitation of the rarely collected species in this clade needs further studies); (ii) the Pacific coast clade including *V. nigricans*, *V. gigantea*, and *V. menziesii*; (iii) *Vicia crocea*, which might best be placed in a monotypic section even though additional work might reveal a sister group relationship to the *V. nigricans* clade.

The results of our phylogenetic analyses show that *Lathyrus saxatilis* is currently included in the wrong genus: the two accessions we have are both placed in the core *Vicia* in our phylogeny and even though our data are not sufficient to pinpoint its exact position (grouping with *V. dumetorum* and *V. americana* in the Bayesian but not in the ML phylogeny) it should certainly be treated as *Vicia saxatilis* (Vent.) Tropea. The taxon was described as *Orobus saxatilis* by Ventenat but interestingly, it was already transferred to *Vicia* in the early 20th century (see Kupicha [8] for details). Kupicha discusses the morphological affinity to *Vicia* (especially *Vicia* sections Vicia and Hypechusa) but although expressing some doubts, the dorsally compressed and adaxially pubescent style led her to include it in *Lathyrus* in a new monospecific section Viciopsis [8].

Regarding the Azorean endemic *V. dennesiana* (Figure 2), we reject Kupicha’s concept of a monotypic section Perditae and find that the species belongs in the Mediterranean clade of section Cracca, where it also fits well based on stylar morphology [7]. The available herbarium specimens for this presumably extinct taxon show a striking overall morphological similarity to the *V. nigricans* group of the American Pacific coasts and Hawaii [9] but our partial DNA sequence data (nuclear ITS and fragments of plastid *matK* from two specimens) reject a close relationship. Kupicha [7] suggested that this species might be a biogeographic link explaining some of the amphi-Atlantic distribution patterns in Vicia but our analyses strongly suggest a single LDD event out of the Mediterranean to the Azores.

Our phylogeny estimates cast some doubt on species concepts applied in the Canary islands and Madeira: the Canarian endemic *Vicia chaetocalyx* is nested in Macaronesian and Mediterranean *V. lutea* accessions and it seems clear that the sequenced material should be assigned to the latter species because they are morphologically almost identical. However, our material came from a recently described population in Gran Canaria [52] and not from the original type material collected by Webb and Berthelot and now in the Florence herbarium, which should be used for further genetic study. A similar problem was reported in a recent study on an endemic bird species from the Cape Verde archipelago [53]. The four Canary and Madeira endemics in the *V. cirrhosa* group show no genetic differences in any of the sequenced regions. Since morphological differences are also minor, a re-evaluation of the group might be necessary (and possibly lumping of all four taxa into *V. cirrhosa*). In contrast, we find a high genetic variability among the relatively few samples included from the *V. sativa* group, where additional Macaronesian endemics might have been overlooked. Lowe’s *Vicia pectinata* for example, has been synonymized with the widespread *V. sativa* but we find three substitutions in the ITS region, which seem to be unique to Lowe’s taxon. Together with some morphological differences and the obvious isolation of the population on an oceanic island, there might be enough evidence to reinstate *V. pectinata*. However, a more detailed study of the *Vicia sativa* group in Macaronesian with several samples per island would be required to properly address this question.

### Biogeographic history of the tribe

The origin of Fabeae is estimated to lie in the Middle Miocene (23–16 Ma). This result, however, could be expected since we constrained the Vicia s.l. crown to 17.5 Ma under a normal prior distribution with standard deviation of 1.9 million years. LDD events (here defined as dispersal across oceans) are relatively common in Fabeae. We reconstruct 12 LDD events over the period of c. 18 million years, which translates into a rate of c. 7 successful LDD events per 10 million years. The same rate was found for Cucurbitaceae [54] but sampling density in that study was lower (c. 25% of the accepted species), so the cucurbit rate is very likely an underestimate.

Migration via Greenland and the North Atlantic land bridge is not an option in Fabeae since the entire tribe is too young (climate cooling made use of that connection very likely impossible some 40 Ma [55]). Migration across the Beringian land bridge is likely for at least one lineage: the ancestors of the Pacific coast species *Vicia nigricans*, *V. gigantea*, and the Hawaiian *V. menziesii*. However, since the lineage later managed to colonize the isolated Hawaiian archipelago, LDD across the Pacific from Asia to the American Pacific coast is also an option or, less parsimonious, LDD from the Mediterranean to the American continent followed by spread to the West coast and extinction in the East. The next three Fabeae lineages, *Vicia* section Australes, *Lathyrus* section Notolathyrus, and the *V. ludoviciana* clade, all reached the American continent in the same time window (9–5 Ma) via LDD from the Mediterranean region across the Atlantic. This suggests favourable conditions for legume seed dispersal in the late Miocene, a ‘window of opportunity’ in the sense of Carine [56]. With the opening of the Bering Strait, c. 5.5-5.4 Ma [57], it became temporarily impossible to migrate to the Americas over land but later reconnections might have allowed terrestrial migration of cold-adapted lineages. For the ancestors of *Lathyrus japonicus*/*L. littoralis*, *L. palustris*, the large New World clade in *Lathyrus* section Orobus, and *Vicia americana* we can thus not decide between terrestrial migration and LDD. More recently, there might have been some transport across the region by Inuit, who use the roasted seeds to prepare drinks [58]. For *L. japonicus* and *L. palustris* the most likely direction is a spread from Asia to North America. In contrast, the *V. americana* lineage might have come the opposite way from North America (*V. americana* s.str., diploid) to Asia (*V. bungei*, tetraploid to hexaploid). Both *V. americana* and *V. bungei* are sister to the European *Vicia onobrychioides* L. and *V. dumetorum* and the ancestral lineage of the latter species might have spread into North America across the North Atlantic.

The now extinct Azorean endemic *Vicia dennesiana* might have reached the Azorean archipelago in the North Atlantic via LDD c. 2 Ma and the Canary islands and Madeira endemics of the *Vicia scandens* clade colonized those archipelagos via LDD about 4 Ma. We can therefore reject the hypothesis of ancient stepping stone dispersal via the Atlantic islands because all the island lineages are relatively recent arrivals compared to the age of Macaronesia (c. 60 million years [43]) and none of the American lineages is nested in an Atlantic island clade. In some cases our age estimates might also be biased old because of extinction events. The *Vicia dennesiana* and *V. scandens* lineages could represent the last survivors of previously more species-rich Eurosiberian clades in Macaronesian refugia and might be even younger than estimated if the Northwest African species of section Cracca were included, for which we failed to obtain material.

### Evolution of life form and floral morphology in Fabeae

Ancestral character reconstruction shows that life form, stylar characters, and chromosome number are of limited value for systematic grouping of Fabeae. Style shape, a traditional character for Fabeae classification [7,8], varies little within the tribe: most species have a dorsiventrally compressed style, which is also reconstructed as most likely ancestral condition in the tribe (Figure 9). The finding that an annual life form is most likely the ancestral state in the tribe is unexpected and rejects earlier hypotheses of multiple evolution of annual life form as an adaptation to range expansion into xeric habitats [59]. It fits, however, with the result of the biogeographic analysis locating the region of origin in the Mediterranean and not in the temperate or boreal forests, where a perennial life form would have been an advantage. A basic chromosome number of 2n=14 has already been suggested by other authors (e.g. [60]), an ancestral, evenly hairy, dorsiventrally compressed style fits with recent results of small-scale character reconstructions [23].

### Origin of legume crops

Our analyses shed light on a group of generally overlooked Mediterranean *Lathyrus* species that apart from the Caucasian *Vavilovia* species might be very interesting for breeding of new *Pisum* varieties due to benefitial traits including drought tolerance and perennial life form: *L. gloeosperma*, *L. neurolobus* Boiss. & Heldr., and *L. nissolia* all have affinities to the *Pisum* plus *Vavilovia* clade and might be included in breeding programs. The placement of *V. faba* far from the other members of section Faba sensu Kupicha [7] and somewhere among *V. oroboides* Wulfen, *V. bithynica* (L.) L., and the *V. sativa-V. sepium* clade (Figure 3; Additional file 10: Figure S8; Additional file 12: Figure S10) is in agreement with earlier morphological, cytological and DNA studies [14,17,18,61]. *Vicia faba* differs in chromosome number from most of the suggested relatives found in our analyses. *Vicia bithynica*, *V. oroboides*, and *V. sepium* all have 2n=14 chromosomes, whereas *V. faba* (and *V. sativa* s.l.) has 2n=12 (Additional file 2: Table S1; Figure 7), which will make crossings for plant breeding purposes difficult. The *Lens* clade has its closest relatives among early branching *Vicia* clades followed by c. 10 million years of independent evolution. While this could be a big obstacle for breeding efforts, the species of the African *V. paucifolia* complex and the Asian *V. subvillosa* might be worth testing if initial screenings reveal benefitial traits. For the *V. sativa* complex, a sister group relationship to *V. pyrenaica* seems very likely and the species should be explored. The high sequence diversity detected among our accessions of *V. sativa* s.l. corresponds to earlier findings of karyological and AFLP studies [62,63] and a detailed revision of the group is recommended in order to detect the most useful growth forms.

## Conclusions

Based on nuclear and chloroplast phylogenies, we conclude that tribe Fabeae evolved in the Eastern Mediterranean in the middle Miocene. Ancestral Fabeae probably were annual plants with a chromosome number of 2n=14, and evenly hairy, dorsiventrally compressed style. From the Mediterranean, the tribe expanded its range at least 15 times into Central and Western Europe and 24 times to Asia, twice to Tropical Africa, and at least seven times across the Atlantic/Pacific to the Americas. The middle-Atlantic islands were colonized four times but did not serve as stepping-stones for lineages colonizing the New World. Our biogeographic analyses show that long distance dispersal events are relatively common in Fabeae (one successful event per 1.5 million years). Current generic and infrageneric circumscriptions in Fabeae do not reflect monophyletic groups and should be revised.

## Methods

### Sampling and DNA extraction

Leaf samples of 262 species of Fabeae were collected in the field and dried in silica gel or taken from herbarium vouchers, 125 of them never sequenced before (Additional file 2: Table S1). We tried to cover all described sections and all biogeographical regions of the Fabeae worldwide distribution. The morphologically most divergent species among the not sequenced taxa is *V. koeieana*, native to the Eastern Mediterranean/Asia Minor. Based on overall morphology, the species is suspected to be close to *V. cypria*, which we have included. Total genomic DNA was isolated from c. 100 mg dry leaf material with commercial plant DNA extraction kits (DNeasy, Qiagen; NucleoSpin, Machery-Nagel), following the manufacturers’ manuals. For the polymerase chain reaction (PCR) we used standard protocols with primer annealing at 48°C for *rbcL*, 49°C for *matK* and *psbA-trnH*, and 52–55°C for the *trnL*, *trnS-trnG*, and ITS1-5.8S-ITS2 regions. Reaction products were purified using ExoSAP-IT (USB Corporation, Cleveland, OH, USA) and cycle sequencing was performed with BigDye Terminator v3.1 cycle sequencing kits on an ABI 3730 sequencer (Applied Biosystems, Foster City, CA, USA) or sent to Functional Biosciences, Inc. (Madison, WI, USA) for sequencing. To amplify *rbcL*, we used the primers *rbcL*-1F, *rbcL*-1460R and the internal primers *rbcL*-600F and *rbcL*-724R [64], for *matK*, we used *matK*-F1, *matK*-1100F and *matK*-1932R [36,37], and for *psbA-trnH* we used the primer pair *psbA* (5^′^- GTT ATG CAT GAA CGT AAT GCT C) and *trnH* (5^′^- CGC GCA TGG TGG ATT CAC AAA TC) by Sang et al. [65]. For *trnL/trnL-trnF*, *trnS-trnG*, and the ITS region, we designed new primer pairs: *trnL*-V (5′-GCC TTG GTA TGG AAA CTT ACC A-3^′^) and *trnF*-V (^′^5-CGA CCA TTC TTG ACG CAC-3^′^), *trnS*-V (5^′^-GAT ACS TCG SAT AAA CAA AAA GAA C-3^′^) and *trnG*-V (5^′^-CAT GTT TCG TAA AGG GCC CCC TAA TG-3^′^), and ITS-VF (5^′^-TCG ATG CCT TAC ATG CAG TG-3^′^) and ITS-VR (5^′^-TAG AAA CGC ATA TGG GTA AAA GAG-3^′^).

### Sequence alignment and phylogenetic analyses

Nine hundred and two sequences were generated for this study and combined with all available sequences from previous studies (mainly [13,23]). Additional file 2: Table S1 lists the relevant taxonomic names with authors, plant sources, and GenBank accession numbers. Sequences from other authors were carefully selected and compared to all available sequence data to avoid including misidentified material. All doubtful sequences were left aside.

Sequences were edited with Sequencher (4.9; Gene Codes, Ann Arbor, Michigan, USA) and Geneious pro v.5.0.4 [66], and aligned using MAFFT [67]; the final alignments were checked visually in MacClade v.4.08 [68]. The aligned nuclear ITS matrix comprised 394 ingroup accessions of 260 species with 668 nucleotides (1.4% gaps and 8.6% missing data); the *rbcL* matrix comprised 79 ingroup accessions of 72 species with 1352 nucleotides and 14% missing data; the *matK* matrix comprised 163 ingroup accessions of 118 species with 1587 nucleotides (0.2% gaps and 28% missing data); for the three spacer regions, we included 191 ingroup accession (146 species) and 733 nucleotides for *trnL-trnF*, 171 ingroup accessions (125 species) with 1174 nucleotides for *trnS-trnG*, and 150 ingroup accessions (111 species) with 688 aligned nucleotides for *psbA-trnH*. The percentages of gaps or missing data were 3% gaps/20% missing data for *trnL-trnF*, 3% gaps/40% missing data for *trnS-trnG*, and 15% gaps/29% missing data for *psbA-trnH*. Twenty-one unambiguous gaps in the ITS matrix, 51 in the *trnS-trnG* matrix, and ten in the *psbA-trnH* matrix were coded as binary characters based on Simmons & Ochoterena [69]. We furthermore combined all plastid regions into one alignment (341 ingroup accessions, 218 species, 5501 nucleotides, 1% gaps/60% missing data) and then combined the plastid and the nuclear ITS datasets into one final matrix with 470 ingroup accessions (262 species), 6200 aligned nucleotides, 1% gaps and 66% missing data. Finally, to obtain a condensed input dataset for the BEAST analyses (see below), we built a combined plastid and ITS matrix with multiple accessions per species merged into a single consensus sequence using the “merge sequence” function in MacClade 4.08 [68], which resulted in 262 ingroup taxa (6274 nucleotides, 1% gaps, 65% missing data).

Maximum likelihood (ML [70]) tree searches and ML bootstrap searches [71] were performed using RAxML 7.0.3 [72]. Based on the Akaike Information Criterion [73] as implemented in jModeltest [74] we selected the GTR + Γ model (six general time-reversible substitution rates, assuming gamma rate heterogeneity), with model parameters estimated over the duration of specified runs. Analyses in RAxML were run on all seven sequence data sets and both with the combined un-partitioned data and with a model that partitioned the plastid data from the ITS data.

Bayesian MCMC inference [75] used the GTR + Γ model (with the default four rate categories) plus a proportion of invariable sites and relied on MrBayes 3.1.2 [76]. Markov chain Monte Carlo (MCMC) runs started from independent random trees, were repeated twice, and extended for five million generations, with trees sampled every 100th generation. We used the default priors in MrBayes, namely a flat Dirichlet prior for the relative nucleotide frequencies and rate parameters, a discrete uniform prior for topologies, and an exponential distribution (mean 1.0) for the gamma-shape parameter and branch lengths. Convergence was assessed using Tracer v. 1.5 [77]. The data matrices and trees have been deposited in TreeBASE (http://www.treebase.org/) study number S13228.

### Molecular dating analysis

To translate genetic distances into absolute times, we used Bayesian time estimation with an uncorrelated-rates model as implemented in BEAST v. 1.7.1 [78]. Since we have been unable to trace any fossils for the tribe, we use four secondary calibration points based on [38] and a normal prior distribution. Specifically, we constrained the root node (the split *Ononis* - rest to 24.7 and standard deviation (SD) of 2.3 Ma, the *Vicia* sensu lato crown age was set to 17.5, SD=1.9, the *Vicia* sensu stricto crown clade to 11.8, SD=1.7 Ma, and the clade comprising *Lathyrus sativus* and *L. latifolius* to 6.3, SD=1.3 Ma. BEAST analyses used the GTR + Γ model with six rate categories. Metropolis coupled Monte Carlo Markov chains were run for 20 million generations, sampling every 1000th generation. Of the 20,001 posterior trees, we excluded the first 2500 as burnin based on convergence assessment using Tracer v. 1.5 [77].

### Biogeographic analyses

To reconstruct the biogeographical history of Fabeae, we coded species’ geographic ranges as an unordered multi-state character, using the following eight character states: (i) Central and Western Europe, (ii) Mediterranean region, (iii) Macaronesian islands, (iv) Asia, (v) Tropical Africa, (vi) North America, (vii) South America, and (viii) Hawaii. We then performed Bayesian ancestral area reconstructions [79] using BEAST v. 1.7.1 and a CTMC model with a gamma prior (shape = 1) and an exponential prior (mean = 1). Otherwise we used the same calibration points and MCMC settings as in the previously described BEAST molecular dating runs.

### Ancestral state reconstruction

To analyse evolution of life form, chromosome number, and stylar morphology, we used the ML tree built from the condensed dataset for the 262 species (Additional file 12: Figure S10). We mapped the evolution of stylar shape, stylar hair patterns, chromosome number and annual versus perennial habit onto the phylogeny by defining four, five, six, and two unordered states respectively (see Additional file 2: Table S1). Information on morphological traits came from personal study of herbarium material and from the detailed lists in [7,8,23,80,81]. To infer ancestral states, we used maximum likelihood as implemented in Mesquite ver. 2.75 [82] (http://mesquiteproject.org/mesquite/mesquite.html). All analyses were carried out on the preferred highest likelihood tree, with branch lengths set to the maximum likelihood values obtained under the GTR + G model (above). Likelihood analyses in Mesquite used the Markov k-state one-parameter model, which is a generalization of the Jukes–Cantor model [83] and assumes a single rate for all transitions between character states. We let Mesquite estimate the transition parameters of the model, based on the tip trait states in the 262-taxon tree and its branch lengths.

## Competing interests

The authors declare that they have no competing interests.

## Authors’ contributions

All authors collected data, HS analyzed data, all authors discussed results and wrote the manuscript. All authors read and approved the final manuscript.

## Supplementary Material

Additional file 1 **Annex 1.** Accepted species of the tribe Fabeae. Overview table for Fabeae with genus, subgenus, and section, distribution and synonyms for each accepted species. Data compiled from [3,7,8] and herbarium label information. Click here for file

Additional file 2 **Table S1.** Material used for phylogenetic analyses. Data for sequences used in the phylogenetic analyses: name, origin, Genbank accession number and voucher specimen for all sequenced taxa and for the sequences retrieved from Genbank. Click here for file

Additional file 3 **Figure S1.** Best ITS maximum likelihood phylogeny of the Fabeae. Best maximum likelihood tree based on the ITS dataset for 394 ingroup accessions (260 species) plus five outgroup species (668 aligned nucleotides). Likelihood bootstrap values ≥ 50% are given at the nodes. Click here for file

Additional file 4 **Figure S2.** Best *rbcL* maximum likelihood phylogeny of the Fabeae. Best maximum likelihood tree based on the *rbcL* dataset for 79 ingroup accessions (72 species) plus five outgroup species (1352 aligned nucleotides). Likelihood bootstrap values ≥ 50% are given at the nodes. Click here for file

Additional file 5 **Figure S3.** Best *matK* maximum likelihood phylogeny of the Fabeae. Best maximum likelihood tree based on the *matK* dataset for 163 ingroup accessions (118 species) plus five outgroup species (1587 aligned nucleotides). Likelihood bootstrap values ≥ 50% are given at the nodes. Click here for file

Additional file 6 **Figure S4.** Best *trnS-trnG* maximum likelihood phylogeny of the Fabeae. Best maximum likelihood tree based on the *trnS-trnG* dataset for 171 ingroup accessions (125 species) plus three outgroup species (1174 aligned nucleotides). Likelihood bootstrap values ≥ 50% are given at the nodes. Click here for file

Additional file 7 **Figure S5.** Best *trnL/trnL-trnF* maximum likelihood phylogeny of the Fabeae. Best maximum likelihood tree based on the *trnL/trnL-trnF* dataset for 191 ingroup accessions (146 species) plus four outgroup species (733 aligned nucleotides). Likelihood bootstrap values ≥ 50% are given at the nodes. Click here for file

Additional file 8 **Figure S6.** Best *psbA-trnH* maximum likelihood phylogeny of the Fabeae. Best maximum likelihood tree based on the *psbA-trnH* dataset for 150 ingroup accessions (111 species) (688 aligned nucleotides). Likelihood bootstrap values ≥ 50% are given at the nodes. Click here for file

Additional file 9 **Figure S7.** Best plastid marker maximum likelihood phylogeny of the Fabeae. Best maximum likelihood tree based on the combined plastid marker dataset for 353 ingroup accessions (230 species) plus seven outgroup species (5501 aligned nucleotides). Likelihood bootstrap values ≥ 50% are given at the nodes. Click here for file

Additional file 10 **Figure S8.** Bayesian consensus phylogeny for the combined dataset. Bayesian phylogeny reconstruction using MrBayes on a combined sequence alignment. When multiple accessions were present in the full dataset, we merged them into one consensus sequence per species resulting in 262 ingroup species (6274 nucleotides). Bayesian posterior probability values ≥ 0.8 are given at the nodes. Major clades (currently accepted genera plus *Ervum* and *Ervilia*) colour-coded according to the system in Figure 3. Click here for file

Additional file 11 **Figure S9.** Chronogram of the Fabeae. Dated phylogeny of the Fabeae estimated using BEAST on a combined sequence alignment. When multiple accessions were present in the full dataset, we merged them into one consensus sequence per species resulting in 262 ingroup species (6274 nucleotides). The age estimates with 95% confidence intervals (green bars) are given at the nodes. Click here for file

Additional file 12 **Figure S10.** Consensus ML phylogeny of the Fabeae. Best maximum likelihood tree based on a combined plastid and ITS matrix with multiple accessions per species merged into a single consensus sequence resulting in 262 ingroup species (6274 nucleotides). Click here for file
