# Supplementary figures and images for: Systematics, biogeography, and character evolution of the legume tribe Fabeae with special focus on the middle-Atlantic island lineages
Source: BMC Evol Biol. 2012 Dec 25;12:250. doi: 10.1186/1471-2148-12-250 (PMC3547781; doi:10.1186/1471-2148-12-250)

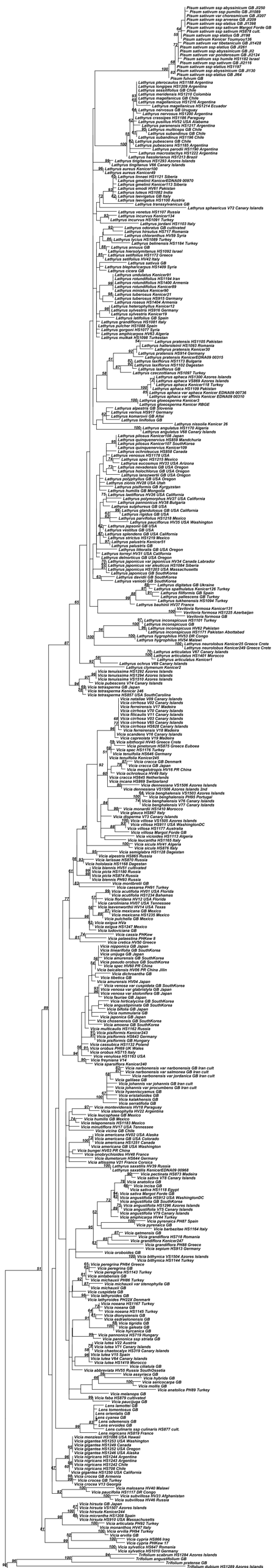

Supplement: Additional file 3 — Figure S1. Best ITS maximum likelihood phylogeny of the Fabeae. Best maximum likelihood tree based on the ITS dataset for 394 ingroup accessions (260 species) plus five outgroup species (668 aligned nucleotides). Likelihood bootstrap values ≥ 50% are given at the nodes. [file 1471-2148-12-250-S3.pdf]

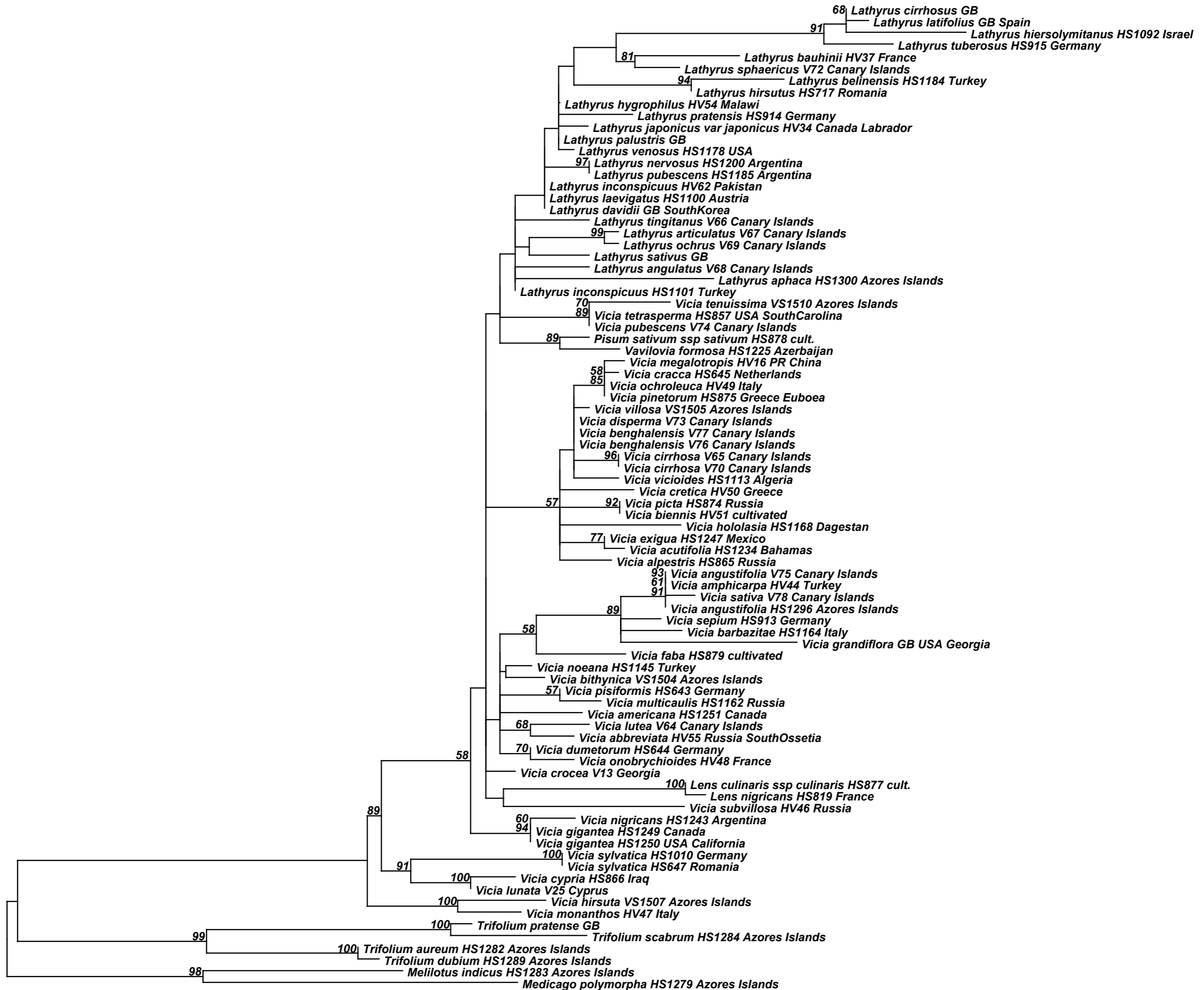

Supplement: Additional file 4 — Figure S2. Best rbcL maximum likelihood phylogeny of the Fabeae. Best maximum likelihood tree based on the rbcL dataset for 79 ingroup accessions (72 species) plus five outgroup species (1352 aligned nucleotides). Likelihood bootstrap values ≥ 50% are given at the nodes. [file 1471-2148-12-250-S4.pdf]

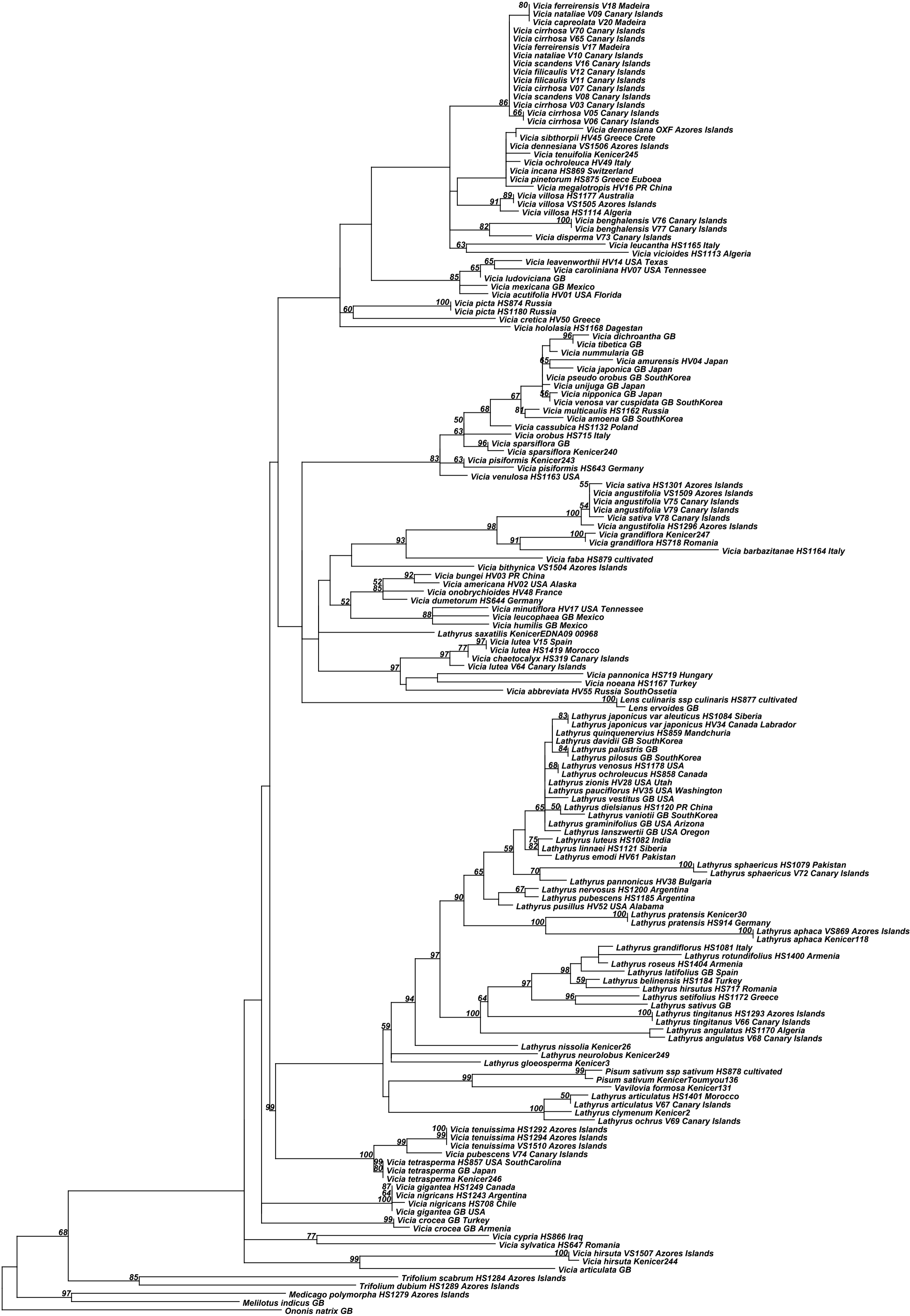

Supplement: Additional file 5 — Figure S3. Best matK maximum likelihood phylogeny of the Fabeae. Best maximum likelihood tree based on the matK dataset for 163 ingroup accessions (118 species) plus five outgroup species (1587 aligned nucleotides). Likelihood bootstrap values ≥ 50% are given at the nodes. [file 1471-2148-12-250-S5.pdf]

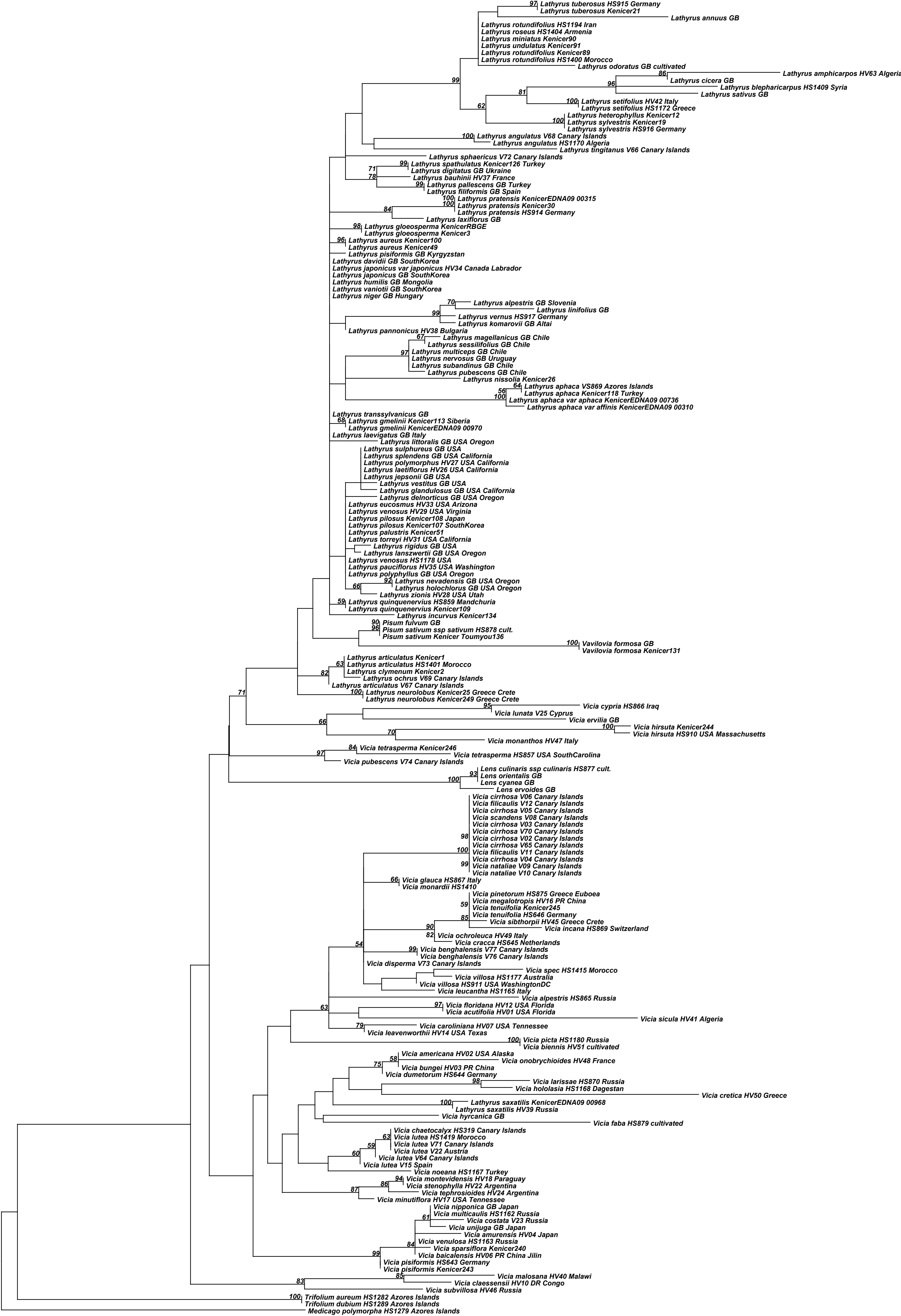

Supplement: Additional file 6 — Figure S4. Best trnS-trnG maximum likelihood phylogeny of the Fabeae. Best maximum likelihood tree based on the trnS-trnG dataset for 171 ingroup accessions (125 species) plus three outgroup species (1174 aligned nucleotides). Likelihood bootstrap values ≥ 50% are given at the nodes. [file 1471-2148-12-250-S6.pdf]

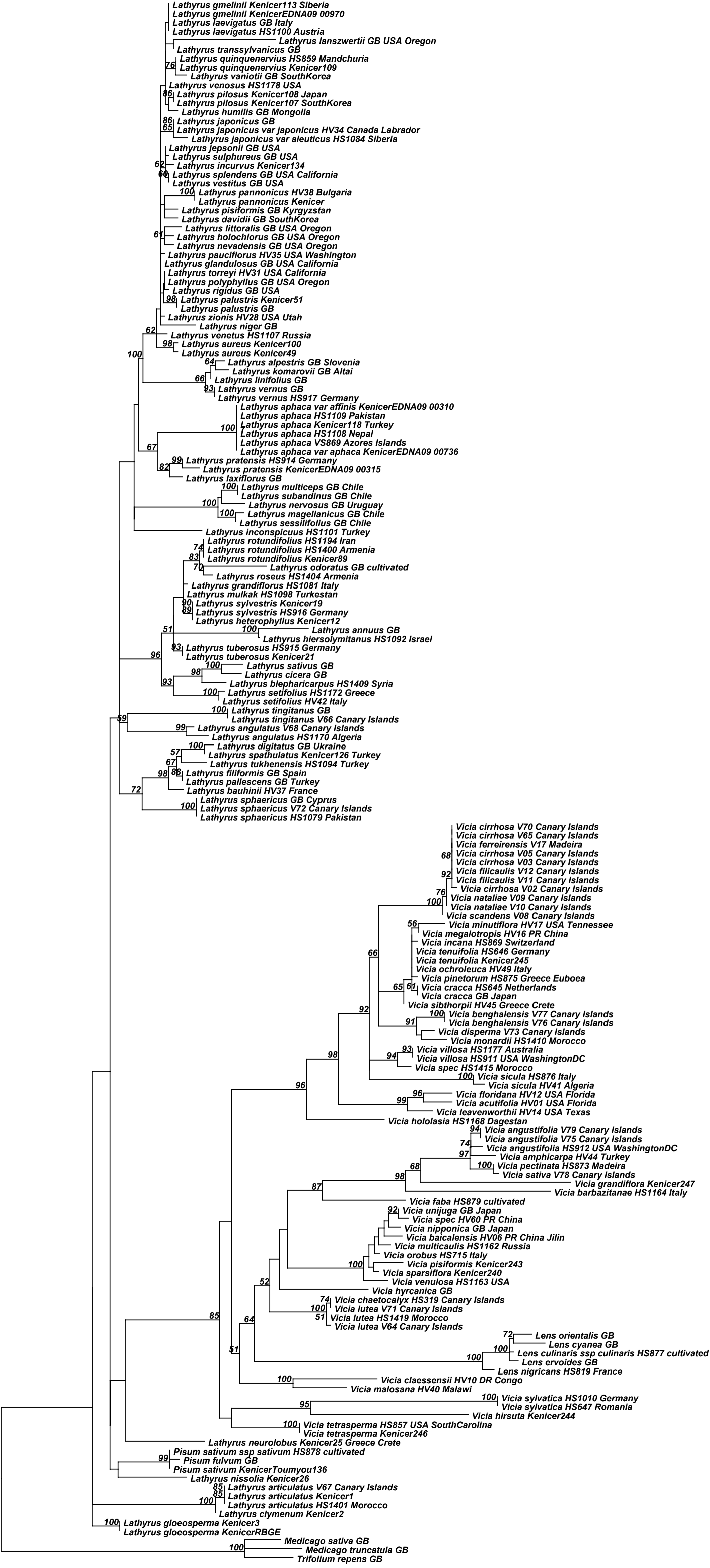

Supplement: Additional file 7 — Figure S5. Best trnL/trnL-trnF maximum likelihood phylogeny of the Fabeae. Best maximum likelihood tree based on the trnL/trnL-trnF dataset for 191 ingroup accessions (146 species) plus four outgroup species (733 aligned nucleotides). Likelihood bootstrap values ≥ 50% are given at the nodes. [file 1471-2148-12-250-S7.pdf]

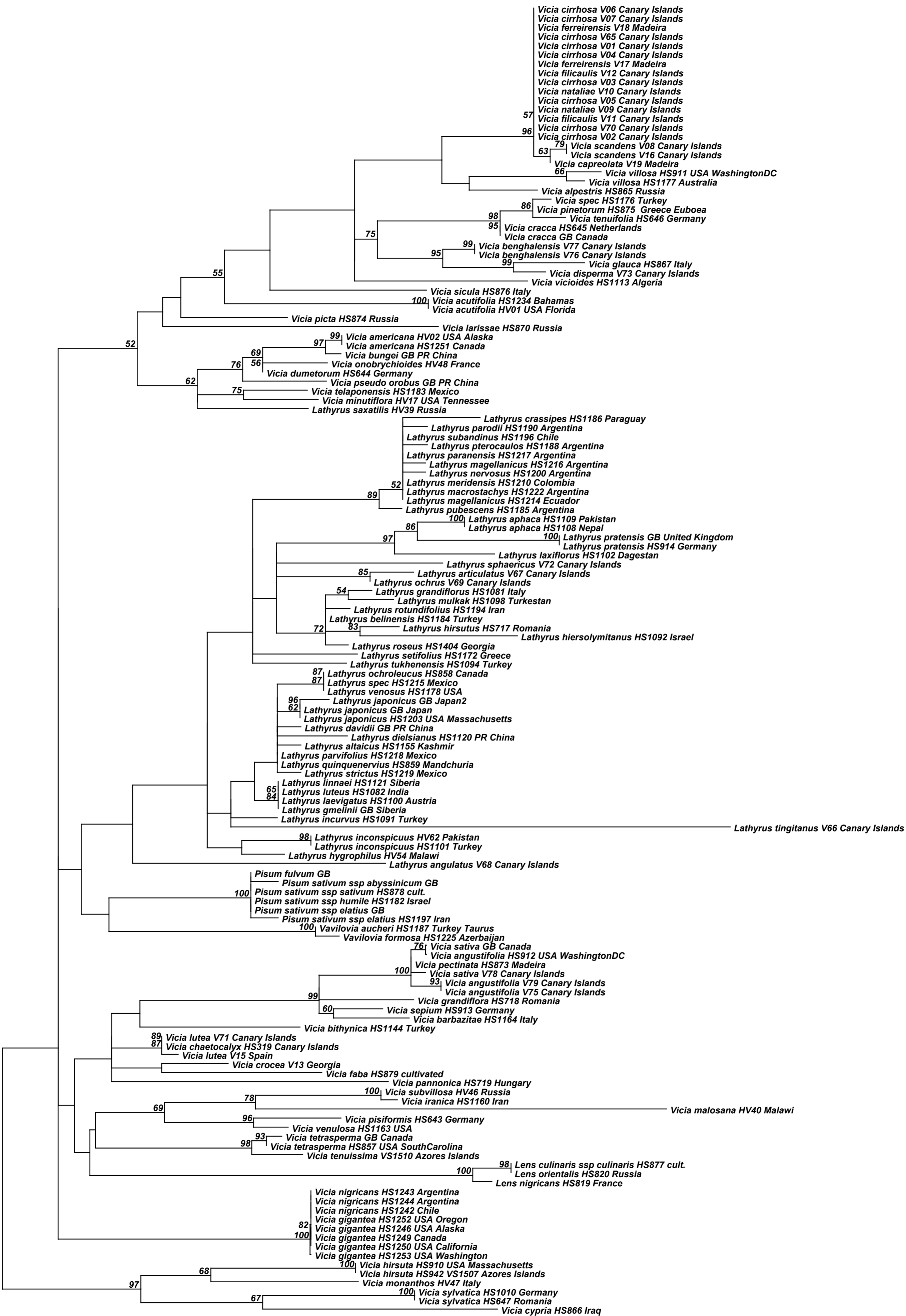

Supplement: Additional file 8 — Figure S6. Best psbA-trnH maximum likelihood phylogeny of the Fabeae. Best maximum likelihood tree based on the psbA-trnH dataset for 150 ingroup accessions (111 species) (688 aligned nucleotides). Likelihood bootstrap values ≥ 50% are given at the nodes. [file 1471-2148-12-250-S8.pdf]

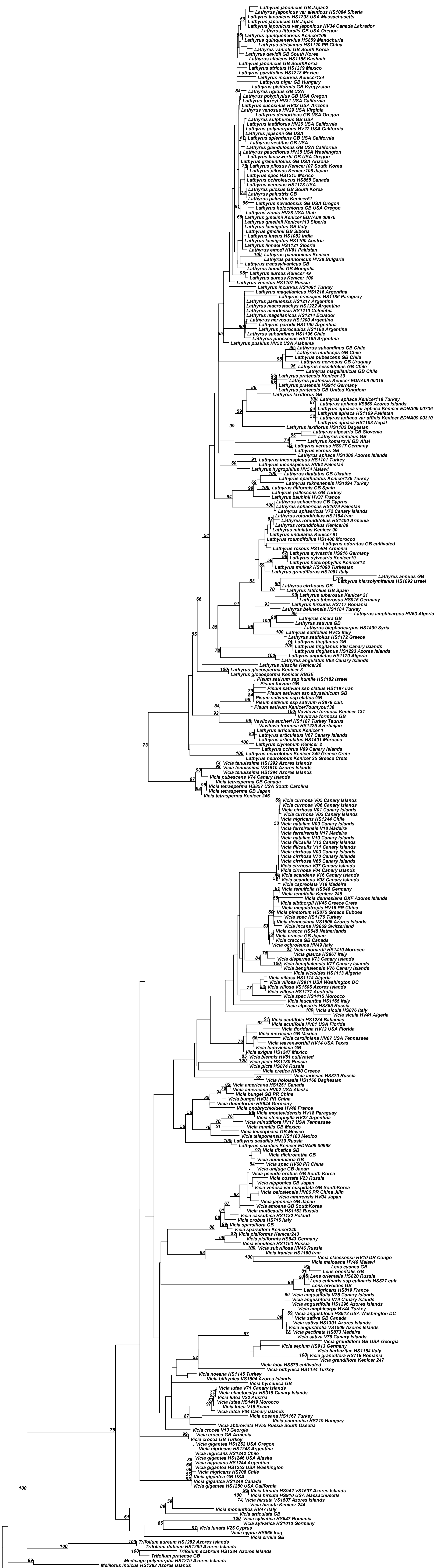

Supplement: Additional file 9 — Figure S7. Best plastid marker maximum likelihood phylogeny of the Fabeae. Best maximum likelihood tree based on the combined plastid marker dataset for 353 ingroup accessions (230 species) plus seven outgroup species (5501 aligned nucleotides). Likelihood bootstrap values ≥ 50% are given at the nodes. [file 1471-2148-12-250-S9.pdf]

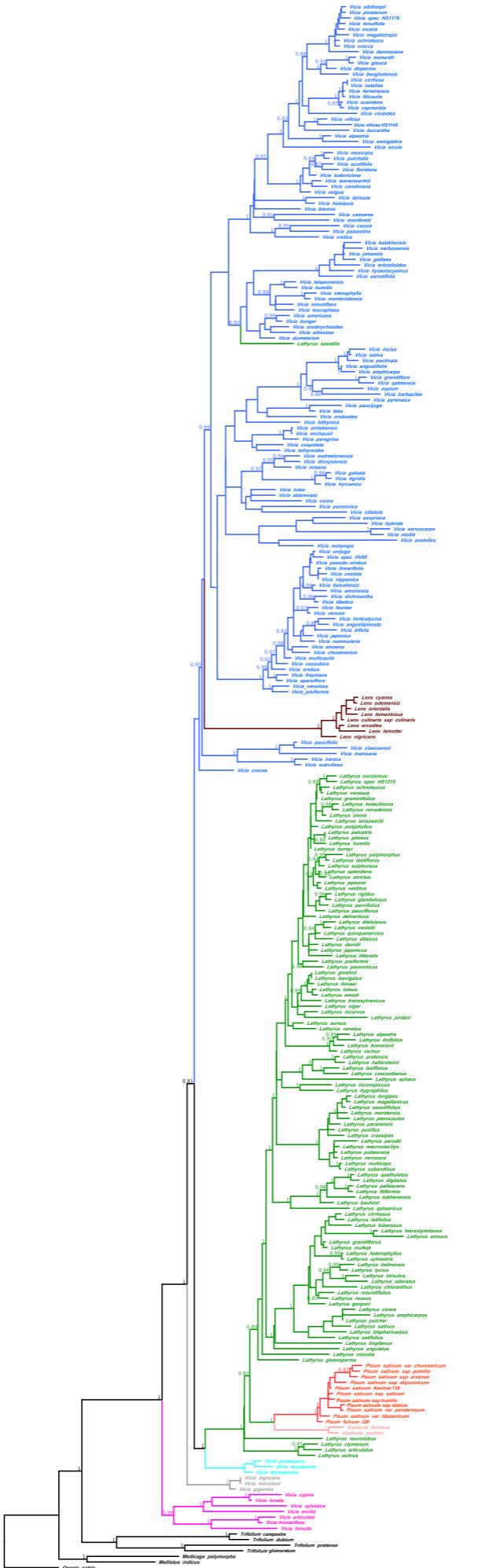

Supplement: Additional file 10 — Figure S8. Bayesian consensus phylogeny for the combined dataset. Bayesian phylogeny reconstruction using MrBayes on a combined sequence alignment. When multiple accessions were present in the full dataset, we merged them into one consensus sequence per species resulting in 262 ingroup species (6274 nucleotides). Bayesian posterior probability values ≥ 0.8 are given at the nodes. Major clades (currently accepted genera plus Ervum and Ervilia) colour-coded according to the system in Figure 3. [file 1471-2148-12-250-S10.pdf]

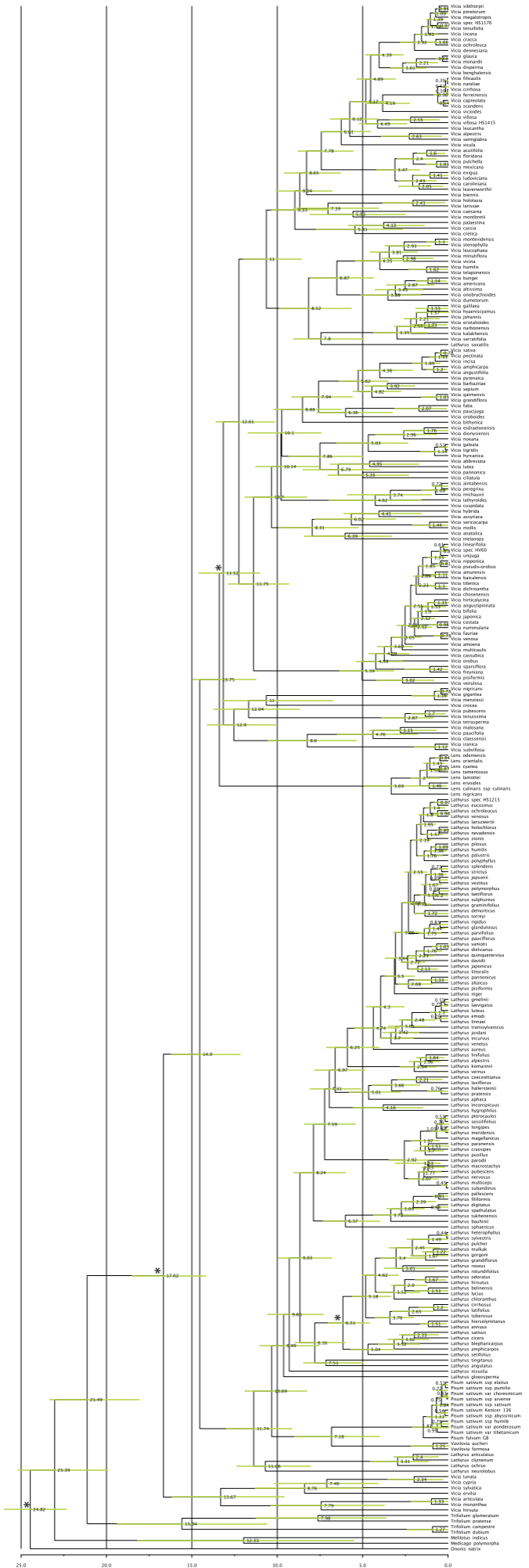

Supplement: Additional file 11 — Figure S9. Chronogram of the Fabeae. Dated phylogeny of the Fabeae estimated using BEAST on a combined sequence alignment. When multiple accessions were present in the full dataset, we merged them into one consensus sequence per species resulting in 262 ingroup species (6274 nucleotides). The age estimates with 95% confidence intervals (green bars) are given at the nodes. [file 1471-2148-12-250-S11.pdf]

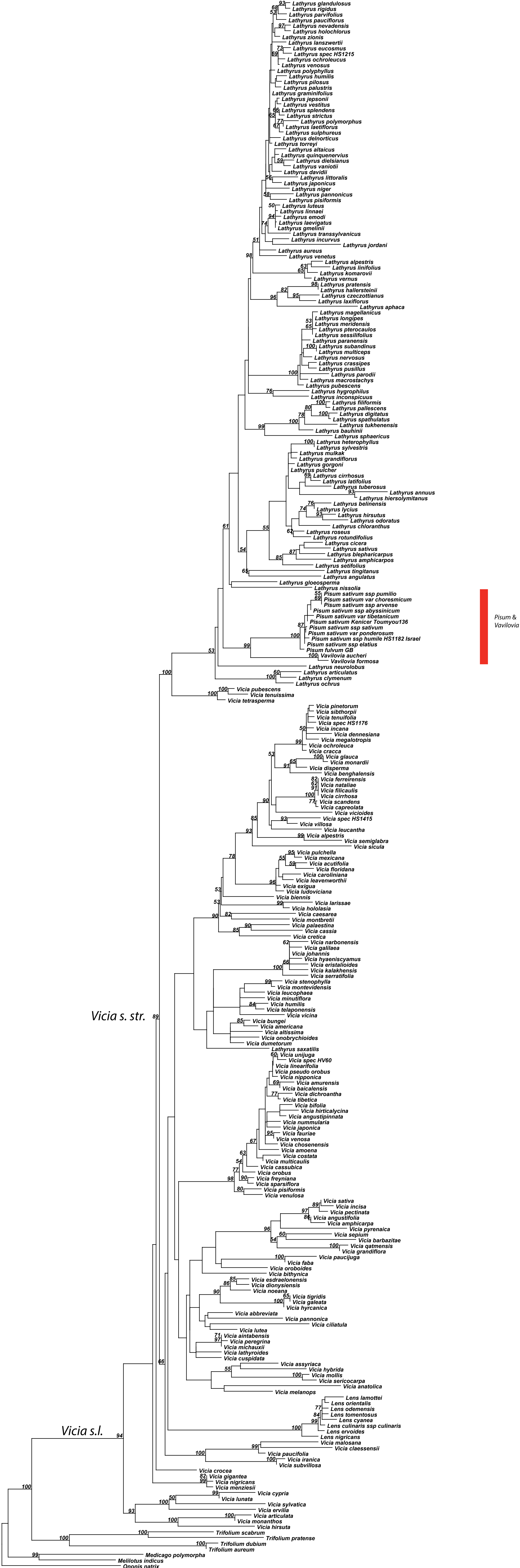

Lathyrus (incl. Pisum/Vavilovia)

Ervum

Vicia (incl. Lens)

Vicia ?

Ervilia

outgroups

Supplement: Additional file 12 — Figure S10. Consensus ML phylogeny of the Fabeae. Best maximum likelihood tree based on a combined plastid and ITS matrix with multiple accessions per species merged into a single consensus sequence resulting in 262 ingroup species (6274 nucleotides). [file 1471-2148-12-250-S12.pdf]
